# Out-of-field effects: lessons learned from partial body exposure

**DOI:** 10.1007/s00411-022-00988-0

**Published:** 2022-08-24

**Authors:** S. Pazzaglia, M. Eidemüller, K. Lumniczky, M. Mancuso, R. Ramadan, L. Stolarczyk, S. Moertl

**Affiliations:** 1 Laboratory of Biomedical Technologies, ENEA CR-Casaccia, Via Anguillarese 301, 00123 Rome, Italy; 2 Institute of Radiation Medicine, Helmholtz Zentrum München, Ingolstädter Landstraße 1, 85764 Neuherberg, Germany; 3 Department of Radiobiology and Radiohygiene, Unit of Radiation Medicine, National Public Health Centre, Albert Florian u. 2-6, 1097 Budapest, Hungary; 4 Radiobiology Unit, Belgian Nuclear Research Centre (SCK CEN), Mol, Belgium; 5 Danish Centre for Particle Therapy, Palle Juul-Jensens Boulevard 25, 8200 Aarhus N, Denmark; 6 Federal Office for Radiation Protection, Ingolstädter Landstr. 1, 85764 Oberschleißheim, Germany

**Keywords:** Cardiovascular disease, Extracellular vesicles, Brain, Radiation dosimetry, Out-of-field doses, Systemic radiation effects, Immune cells, Secondary cancer

## Abstract

Partial body exposure and inhomogeneous dose delivery are features of the majority of medical and occupational exposure situations. However, mounting evidence indicates that the effects of partial body exposure are not limited to the irradiated area but also have systemic effects that are propagated outside the irradiated field. It was the aim of the “Partial body exposure” session within the MELODI workshop 2020 to discuss recent developments and insights into this field by covering clinical, epidemiological, dosimetric as well as mechanistic aspects. Especially the impact of out-of-field effects on dysfunctions of immune cells, cardiovascular diseases and effects on the brain were debated. The presentations at the workshop acknowledged the relevance of out-of-field effects as components of the cellular and organismal radiation response. Furthermore, their importance for the understanding of radiation-induced pathologies, for the discovery of early disease biomarkers and for the identification of high-risk organs after inhomogeneous exposure was emphasized. With the rapid advancement of clinical treatment modalities, including new dose rates and distributions a better understanding of individual health risk is urgently needed. To achieve this, a deeper mechanistic understanding of out-of-field effects in close connection to improved modelling was suggested as priorities for future research. This will support the amelioration of risk models and the personalization of risk assessments for cancer and non-cancer effects after partial body irradiation.

## Introduction

Partial body exposure and inhomogeneous dose delivery are features of the majority of medical and occupational exposure situations. In the medical field partial body irradiation is the mainstay for therapeutic as well as for diagnostic applications. Even in accidental exposure scenarios the initial dose deposition may be distributed in-homogenously due to partial shielding. However, in the meantime there is substantial evidence that the response to partial body irradiation pertains to the whole organism. Radiation effects are not restricted to the irradiated area, but also occur in lower exposed surrounding tissue and in non-irradiated neighboring or distant tissues which considerably extends the radiation response (summarized as out-of-field effects) (Pouget et al. 2018; Nikitaki et al. 2016). Therefore, the exact quantification of dose distribution patterns and the understanding of radiation-induced biological mechanisms in out-of-field areas is required for the identification of potential health consequences. This knowledge is critical for both, the evaluation of radiation effectiveness in radiotherapy and diagnostic application as well as for radiation risk assessment.

To acknowledge the relevance of this topic and to foster research in clinical/epidemiological/dosimetric and biological/mechanistic aspects in the field of out-of-field effects, the MELODI (Multidisciplinary European Low Dose Initiative) and EURAMED (European Alliance for Medical Radiation Protection Research platform) platforms included this topic into its strategic research agendas. Furthermore, projects, concerning the effects of partial body irradiation, particularly out-of-field effects, and how they may affect health risk following exposure, were recently funded from the Euratom Research and training programme 2014–2018, in the framework of the CONCERT EJP [grant agreement No 662287]. LEU-TRACK (https://concert-h2020.eu/sites/concert_h2020/files/uploads/Deliverables/D9/Leu-Track/_Lists_Deliverables_Attachments_215_D9.99_Final-report-of-the-LEU_TRACK-project_approved29052020.pdf) investigated the role of micro-vesicles and their ‘cargo’ in radiation leukemogenesis and SEPARATE (https://sites.google.com/view/separate-project/home) explored the systemic effects of partial-body exposure through a multiomic approach, and how they may affect health risk following exposure.

The classical model in radiation biology in which cells need to be traversed by radiation to be affected (targeted radiation effects) has been challenged by numerous studies, in which cells that were not directly hit displayed radiation-triggered response patterns (non-targeted radiation effects) (Pouget et al. 2018; Morgan and Sowa 2015). Such non-targeted effects include short range interactions between neighbouring cells as well as long range interactions between distant tissues. In vitro as well as animal studies evidenced communication between irradiated and non-irradiated cells, involving the exchange of molecular signals through soluble secreted factors (Wang et al 2018). Small proteins, lipids, second messengers and also DNA and RNA molecules are among the clastogenic factors secreted from irradiated cells that in turn affect bystander cells. These factors can be individually released from irradiated cells, carried in extracellular vesicles or transported via intercellular channels (Hei et al. 2008; Morgan and Sowa 2015; Szatmári et al. 2017). Commonly, components of the immune system were recognized as important mediators of systemic radiation effects (Formenti and Demaria 2009).

Determining the contribution of out-of-field effects in the clinics or generally in vivo is highly challenging and the current knowledge is very limited. Acute effects of local radiotherapy are fatigue, diarrhea and weight loss (Berkey 2010). Secondary cancer and cardiovascular diseases are important long-term out-of-field effects (Mazonakis and Damilakis 2021; Belzile-Dugas and Eisenberg 2021). As the long-term survival of RT patients constantly improves, the understanding of these effects is getting more and more important.

The MELODI Workshop 2020 on the “Spatial and temporal variation in dose delivery” brought together experts in clinical/epidemiological/dosimetric and biological/mechanistic aspects of research. It was organized in four sessions, addressing (1) dose-rate effects, (2) radiation quality, (3) internal exposure and (4) partial body exposure, each generating at least one thematic manuscript (Lowe et al. 2022; Baiocco et al. 2022; Boei et al. 2022; Li et al. 2022). Due to COVID19 outbreak, the workshop was successfully held on-line. Based on their recent work and specific expertise on the topic, the speakers of the partial body exposure sessions were invited to specifically present in this manuscript the current knowledge on the effects of partial body radiation exposure from the clinical, epidemiological and biological point of view not covered by the other manuscripts. The current paper does not intend to represent a summary of the available literature, but rather aims at summarizing the results of the workshop, updated with results of research published since then.

## Out-of-field effects: clinical context

### Radiation dosimetry for radioepidemiological studies in external beam radiotherapy

Studies of dose–response relationship on radiotherapy patient cohorts may potentially help in a limitation of unnecessary healthy tissue exposures, and optimization of treatment techniques. Moreover, as discussed by Harrison (Harrison 2017), radiotherapy patient cohorts, characterized by large population size and dose range covering most of the dose-risk curve, are important in general studies of radiation effects on humans. Such studies are aided by the fact that doses received by patients undergoing radiotherapeutic procedures are well documented and records are typically available years after treatment. One of the most important concerns with respect to non-target doses delivered to healthy tissues surrounding a planning target volume (PTV) is the risk of second primary cancer (SPC) development (Kry et al. 2017). Second primary cancer as defined by Cahan et al. (1948) and adopted by Xu et al. (2008) is the cancer that occurs in locations irradiated in radiotherapeutic procedures, with histology different from that of the original tumour, so it cannot be a metastasis. It appears after typically several years of latency. The second primary cancer should not be present during radiation treatment and the patient should not have a cancer-prone syndrome. NCRP Report 170 (2011) reviews epidemiologic data on SPCs after radiotherapy of the growing number of cancer survivors worldwide. The report proves a clear increase in the risk of SPCs following non-target doses both from primary beam and secondary and scattered radiation. Furthermore, NCRP Report 170 highlights the importance of quantitative estimates of SPC risk. This requires estimation of doses to organs outside the PTV as uncertainties in dose reconstruction may lead to a misunderstanding of the risk of radiation-induced late effects, including SPC risk (Vu et al. 2017). The increasing knowledge about both target and non-target radiation distributions in patients undergoing radiotherapy is an advantage that should be used especially in radio-epidemiological studies with thousands of patients (Howell et al. 2012).

The main sources of radiation during radiotherapy are therapeutic beams and secondary and scattered radiation. Additionally, before and during the course of treatment, the patient may undergo a number of procedures using ionising radiation. Amongst diagnostic procedures, the most common are computed tomography (CT), positron emission tomography (PET) and single-photon emission computerized tomography (SPECT). Nowadays, each patient receives at least one CT scan for dose planning. Finally, all currently used treatment procedures require on-board imaging during patient positioning, which can be performed with 2D kV and MV imaging systems, CT on rails or cone-beam CT scanning (CBCT) (Ding et al. 2018). The number of image-guided verification procedures depends on the particular clinical case as well as the clinical protocol and treatment technique used and can vary considerably (Ding et al. 2018). Furthermore, treatment may be a combination of external beam radiotherapy, brachytherapy and radionuclide-based nuclear medicine procedures (Harrison 2017). In current clinical practice, radiation doses from diagnostic and radiotherapeutic procedures are recorded separately. Moreover, there is a lack of scientific publications reporting the combination of doses from diagnostic, imaging and radiotherapy procedures (Harrison 2017). In general, non-target doses from radiotherapy decrease with the distance from the PTV over a range of tens of Gy to less than 1 mGy (Xu et al. 2008), whilst concomitant doses from diagnostic and imaging procedures fairly uniformly cover the PTV and surrounding tissues with effective doses up to about 30 mSv for multi-slice CT (4DCT) and up about to 130 mSv for pelvis daily kV CBCT for 30 fractions (Halg et al. 2012). In this chapter, we focus on the dosimetry of non-target doses from primary and secondary radiation for radio-epidemiological studies in external radiotherapy.

The primary therapeutic beam covers the target area with uniform and conformal doses at the level of 40–62 Gy delivered in 15–32 fractions (https://www.rcr.ac.uk/publication/radiotherapy-dose-fractionation-third-edition). At the same time, the dose distribution from primary radiation at the border between the PTV and healthy tissues is characterised by strong gradients changing from tens of Gy to a few Gy over a few centimeters. In general, dose distributions from the primary beam depend on the treatment protocol, radiation quality (photons, electrons, protons and heavy ions), effective energy, treatment technique [3-Dimensional Conformal Radiation Therapy (3D CRT), Intensity-Modulated Radiation Therapy (IMRT), Volumetric Arc Therapy (VMAT), Tomotherapy, Stereotactic radiosurgery/stereotactic body radiotherapy (SRS/SBRT), Flattening Filter-Free IMRT, passive scattering or active scanning ion therapy] and treatment planning protocol. Modern radiation techniques such as VMAT can achieve highly conformal dose distributions and improved target volume coverage compared with conventional RT techniques because of continuous rotation of the radiation source and beam intensity modulation. With the additional rotational degrees of freedom, high dose peaks in healthy tissue can be reduced. At the same time, the resulting organ dose distributions in healthy tissue can differ from traditional techniques like 3D-CRT, and mean organ doses can increase due to potential increased body traversal of multiple beams (Xu et al. 2008; Kry et al. 2017).

Secondary and scattered radiation is produced in the interactions of the primary beam with the treatment nozzle and patient body and creates a low dose envelope outside the treatment field. Depending on the quality of primary radiation, scattered and secondary radiation is a mixture of scattered X-rays, secondary γ radiation, neutrons, charged particles, characteristic X-rays, bremsstrahlung radiation and residual radiation from radioactivation (Xu et al. 2008; Kry et al. 2017).

For photon beams, the contribution to out-of-field doses arises from scattered radiation generated within the patient’s body, leakage from the linear accelerator head and scattered radiation produced by the collimators and beam modifiers on the path of the primary beam (Mazonakis and Damiliakis 2021). Non-target doses depend on: field size, number of Monitor Units (MU) which increases collimator scatter (Ruben et al. 2011), accelerator type, beam energy (Brody et al. 2013), treatment technique and finally patient specific beam modifiers (Kry et al. 2017). The radiation spectrum is dominated by scattered photons which undergo single or multiple Compton interactions (Knezevic et al. 2013). For primary beam energies higher than a threshold for photo-neutron interactions [6–13 MeV for most materials (Xu et al. 2008)], secondary neutrons are created with a fast neutron peak between 0.1 and 1 MeV and low-energy tail from neutrons being elastically scattered (Kry et al. 2017). In conventional photon therapy, the out-of-field dose decreases rapidly as the distance from the field edge increases (Miljanić et al. 2013; Di Fulvio et al. 2013).

In proton radiotherapy, the main sources of out-of-field doses are the beam forming elements, both inside the nozzle and close to a patient (collimator, range shifter, energy modulator, compensator), and the patient body. The secondary radiation field is dominated by secondary neutrons with two high-energy peaks in their energy spectrum: first from evaporation and neutron scattering and second from direct (nucleon–nucleon) reactions (Stolarczyk et al. 2018). Secondary neutrons together with secondary photons and scattered and secondary protons create a complex mixed radiation field, which depends strongly on delivery technique (active or passive) (Halg and Schneider 2020), individual patient beam modifiers (Wochnik et al. 2021) and treatment field parameters such as energy and field size or modulation (Mojzeszek et al. 2017). In passive proton therapy, the primary proton beam interacts mostly with components in the nozzle, such as the scatterers and collimators, whilst in active scanning the beam interacts mostly with the patient’s body. Therefore, the neutron out-of-field dose from passively scattered proton radiotherapy is substantially (the order of magnitudes) higher compared with the Pencil Beam Scanning (PBS) technique (Halg and Schneider 2020). For both passive and active proton radiotherapy techniques the out-of-field doses are the highest close to PTV and decrease with increasing distance from PTV (Stolarczyk et al. 2018; Halg and Schneider 2020).

Comprehensive reviews of doses from secondary and scattered radiation were published by Xu et al. (2008); Kry et al. (2017); Halg and Schneider (2020); Mazonakis and Damiliakis (2021). All showing that comparison of out-of-field doses estimations from different studies is a demanding task. Large discrepancies between reported values are among others due to the differences in delivery systems, set-ups and used dosimetry techniques. EURADOS Working Group 9 (Radiation Dosimetry in Radiotherapy) performed a systematic investigation on out-of-field doses in photon and proton radiotherapy for paediatric patients. In all cases investigated by WG9 out-of-field organ doses were assessed inside anthropomorphic phantoms for the same target (6 cm diameter spherical PTV with the center on the left anterior side of the head). This work was recently summarized by Knezevic et al. (2022). Table 1 presents example data for out-of-field dose levels estimated by EURADOS WG9 for 3D-CRT, IMRT (Majer et al. 2017) and Intensity Modulated Proton Therapy (IMPT) with PBS technique (Knezevic et al. 2018). They are compared with organ out-of-field doses simulated for proton passive scattering technique for a spherical 5.6 cm diameter tumour located at the centre of the brain (Sayah et al. 2014). Out-of-field doses for proton PBS therapy are lower when compared 3D-CRT, IMRT and proton passive scattering techniques. The difference is at the level of one order of magnitude close to the brain and more than two orders of magnitude for distal organs. As summarized by Kry et al. (2017) when comparing 3D-CRT with IMRT, IMRT usually offers better conformity (lower doses) near the edge of the PTV but higher doses in distal organs (due to the increased collimator scattering and head leakage). However, it depends strongly on plan parameters, optimization routine, target size and location. As a result, out-of-field doses for the same technique can vary up to two orders of magnitude between different studies (Kry et al. 2017).

**Table 1:** Total organ out-of-field doses (mSv/Gy) for a brain target in a 10-year-old phantom

	Thyroid	Liver	Testes
3D-CRT (6 MV photon)	3.26	0.58	0.17
IMRT (6 MV photon)	2.7	0.58	0.19
Proton passive scattering technique	1.97	0.63	0.30
Proton active scanning technique	0.42	0.03	0.01

For 3D-CRT, IMRT and proton active scanning radiotherapy data are adapted from Knezevic et al. (2022). For passive scattering proton radiotherapy data are adapted from Sayah et al. (2014) assuming 10% contribution from secondary photons to total organ equivalent dose.

The estimation of doses from secondary and scattered radiation for radio-epidemiological studies can be performed with treatment planning systems (TPS), Monte Carlo (MC) modelling, analytical models of dose distribution outside PTV and finally in-phantom measurements.

The method most commonly used for organ dose assessment is dose calculation with a Treatment Planning System (TPS) (Zhang et al. 2015). TPSs give precise dose information for a target and its proximity, typically in the dose range between 0.1 and 60 Gy. Both organ average doses and dose-volume information are available. However, calculations are possible only in the area covered by a planning CT. Older versions of the calculation algorithms give less precise results and in general the calculation accuracy outside the treatment field is reduced. The underestimation of out-of-field doses by TPS increases with distance from the PTV and can reach up to 70% in distal organs (Majer et al. 2017, 2022). Howell et al. (2010) proposed to use TPS for out-of-field organ dose calculation only within 5% isodose. In TPS out-of-field doses from scattered and secondary radiation and doses from imaging and diagnostic procedures are not taken into account. Moreover, the relative biological effectiveness (RBE) of a radiation, important especially for charged particles and neutrons, is not fully considered (Kry et al. 2017).

Monte Carlo modelling is considered the most flexible method of simulating particle interactions within a medium. It allows, for example, for scoring the dose from different particles or interactions separately, and calculations in whole-body computational phantoms (Park et al. 2021). Long computation times and lack of precise information needed to build a proper model of a treatment machine very often prevent wider use of this method (Kry et al. 2017). Moreover, De Saint-Hubert et al. (2022a, b reported a significant dependence of the calculated neutron fluence spectra on the codes used. It has an important implication on both the calculated neutron out-of-field dose and calibration of neutron detectors used for out-of-field dose measurements. Finally, experimental validation of the MC model not only for primary radiation but also for secondary and scattered radiation calculation is required (De Saint-Hubert et al. 2022a, b).

Analytical models, validated by experimental data, may successfully describe both primary beam and out-of-field scattered and secondary radiation, also in a wide range of irradiation conditions. Accuracy of the models is within 30% although much larger errors are possible (Kry et al. 2017). Newhauser et al. (2018) in his review of analytical models of secondary radiation in photon and proton radiotherapy, not only summarised and compared existing models, but also highlighted the need for their further development using precise experimental data.

In-phantom measurements are a gold standard for secondary and scattered radiation dosimetry in radiotherapy. However, providing high quality experimental data in a mixed field of primary, secondary and scattered radiation is challenging. This is mostly due to difficulties in measurements in the dose gradient (averaging effect of detector size and precision of detector positioning), and sensitivity of detectors to different radiation types and different radiation LET (Stolarczyk et al. 2018). Moreover, experiments should be performed in a well-defined and well-described standardised condition (phantom size, target location, target size) to allow comparison between different delivery techniques and connection with clinical cases (Majer et al. 2017; Hauri and Schneider 2019). Robust calibration of detectors and correction of their readouts depending on the radiation quality is essential (Knezevic et al. 2013).

Even though a number of experimental and computational studies on out-of-field doses both for photon and proton radiotherapy is available in the literature, generalisation of the results, which would allow the prediction of out-of-field doses for individual patients, remains a challenge (Kry et al. 2017). Another challenge lies in the estimation of total dose including not only dose from radiotherapy (primary and secondary radiation) but also imaging procedures (Ruhm and Harrison 2020). Different components of total dose received by a patient are characterized by different radiation quality, different energy spectra and different LET distributions (Stolarczyk et al. 2018). Currently, quantities such as effective dose, dose equivalent or ambient dose equivalent typically used for radioprotection are used to sum up contributions from radiotherapy beams, out-of-field radiation and imaging procedures (Kry et al. 2017). However, radioprotection quantities do not take into account fractionation as well as simultaneous irradiation with high doses in a restricted PTV volume and low doses to the rest of the patient body. Moreover, dose equivalent and effective dose use radiation and tissue weighting factors which are concepts developed for radioprotection, where a person is exposed to low doses. Diallo et al. (2009) has shown that 66% of second primary malignant cancer develops in the proximity of the PTV in the area of the primary beam edge dose gradient where also out-of-field doses are the highest. Moreover, this area is typically irradiated during diagnostic and positioning procedures, which also contribute to the total dose (Harrison 2017). Only 22% of second primary malignant cancer is developed at a distance greater than 5 cm from the edge of the irradiated volume in the low dose area from scattered radiation and imaging procedures (Diallo et al. 2009). Therefore, from the radio-epidemiological point of view, it is crucial to estimate dose comprehensively in the proximity of PTV. Another important issue in this area is the presence of dose gradients where radiation is not uniformly distributed. As a consequence, reporting only average organ dose may be misleading (Schneider and Walsh 2017). Part of the organ may be irradiated with a high dose while part may be exposed to lower doses in the primary beam penumbra. Schneider et al. (2005) proposed the concept of an organ equivalent dose (OED) for radiation-induced cancer. It assumes that different dose distributions within an organ are equivalent and correspond to the same OED if they cause the same radiation-induced cancer incidence. For low doses distant from the PTV, the OED is equal to the mean organ dose, while for high doses in the proximity of PTV, it is different from the mean organ dose, because of cell sterilisation effects at high doses.

In summary, development of modern dosimetry tools which would allow for dose estimation in organs covered with inhomogeneous mixed radiation fields is needed to minimise uncertainties in radio-epidemiological studies. Currently available dosimetry methods should be used with consideration of their limitations. As a minimum, a detailed description of assumptions and quantities used should be provided.

### Risk of radiation-induced second primary cancer and heart disease after breast cancer radiotherapy

While radiotherapy plays an important part in the treatment of cancer by reducing the risk of recurrence and improving survival, it also induces unavoidable radiation exposure in the surrounding tissues. Epidemiological studies demonstrated that radiotherapy, despite its tumor killing prosurvival functions, increases the risk of second primary cancer and non-cancer diseases later in life due to the radiation exposure. These health risks become increasingly relevant with improved cure rates of the primary malignancy and prolonged survival. For example, for breast cancer the 10-year relative survival rate in Germany is above 80%, and can even be substantially higher for patients with good tumor status (RKI 2022).

Characteristics of radiotherapy exposures are the huge dose ranges and extreme dose gradients. For breast cancer RT, in organs close to the treatment area, the doses can range from 50 Gy or more down to a few Gy or even below 1 Gy. As discussed in the previous section, modern techniques such as VMAT can lead to different dose distributions than conventional 3D-CRT techniques with unknown consequences for late health risks. Furthermore, the new techniques can increase mean organ doses (Corradini et al. 2018) while also the more distant organs can substantially contribute to secondary cancer risk (Simonetto et al. 2019a).

The strong inhomogeneity and variability of the exposures make assessments of long-term risks difficult. While modern techniques offer the flexibility to adapt the radiation fields, it is often not possible to select among different treatment options regarding long-term risks because of the difficulties to assess the risks. In the high dose region^1^ above about 4 Gy, long-term risks after radiotherapy were assessed by several studies (NCRP 2011). For some cancer sites, such as lung and breast cancer, a dose–response relationship consistent with a linear dose dependence was observed.

However, in the presence of high dose gradients a small variation in the location where the tumor originated can lead to significant changes in dose. It was shown by Schneider et al. (2018) that already the uncertainty of exact tumor location could result in inference of a linear dose–response relationship in epidemiological studies even if the actual dose–response relationship was non-linear. For other cancers, such as leukaemia or thyroid cancer, the data indicated a flattening or downturn at higher doses. However, these high-dose studies usually have limited statistical power at doses below 1–4 Gy. Since parts of the organs close to the treatment area are in the low- and medium dose range, as well as the more distant organs, additional information from other studies is needed. The most informative study is the cohort of the atomic bomb survivors of Hiroshima and Nagasaki (LSS, Life Span Study) which provides robust organ-specific estimates of radiation risk including age dependencies (Preston et al. 2007; Grant et al. 2017). While risk estimates from RT studies are usually derived from treatments using fractionation, the cohort members of the LSS had a single unfractionated exposure. Although no epidemiological data exist that could estimate the influence of fractionation on late health risk, it can be expected that fractionation has less relevance for risk in the low- and medium dose range (Simonetto et al. 2021). Moreover, similar to RT, the atomic bomb survivors were exposed dominantly by high-energy photons at high dose rates.

Comparing the risk coefficients from radiotherapy studies and the LSS, large differences were found for some cancers, in particular for cancers of the lung, breast, and for leukaemia (NCRP 2011; Berrington de Gonzalez et al. 2013). In breast cancer radiotherapy, even for organs close to the treated breast, large organ parts are exposed to low- and medium doses (Joosten et al. 2013). This raises the question whether risk coefficients from radiotherapy studies based on conventional 3D-CRT or older electron techniques can be directly transferred to modern applications like VMAT that may deliver substantially different dose distributions.

It is plausible that risk coefficients from low- and medium doses and from high doses may be different since the radiation-induced biological mechanisms are different. At high doses, the processes of cell killing and repopulation between different fractions become increasingly relevant (Sachs and Brenner 2005). Shuryak and collaborators (2009a; b) developed a mathematical model that considers initiation, inactivation and repopulation of normal and premalignant stem cells during and after radiotherapy. The processes of cell killing and repopulation were also implemented in the model by Schneider (2009) to estimate the dose response and cancer risk for fractionated radiotherapy.

To construct a dose–response relationship for the full relevant dose range based on radiation-epidemiological data, Simonetto et al. (2021) used information both from high dose studies and from the LSS. For lung cancer, breast cancer and leukaemia an intermediate dose range was defined where the excess relative risk was interpolated between the low- and high dose regime. This resulted in a combined, in general non-linear dose response. Organ cancer risks were then estimated by integration of the dose–response relationship over the organ dose distribution. These models were also implemented into the PASSOS software tool to estimate age-integrated risks together with associated uncertainties for various radiotherapy techniques (Eidemüller et al. 2019; PASSOS 2022).

Besides second primary cancer, the risk of late heart disease is a major concern in breast cancer radiotherapy applications. A highly relevant study is the work by Darby et al. (2013) that analysed the risk of ischemic heart disease. It was found that the rates of major coronary events increased linearly with the mean dose to the heart by 0.074 per Gy. These results were based on a case–control study including 2168 women who underwent radiotherapy for breast cancer between 1958 and 2001 in Sweden and Denmark. Recently, Laugaard Lorenzen et al. (2020) re-analysed the Danish part of this data set with an extended follow-up and refined dose estimates. Focussing on the group with tangential photon techniques and precise dose estimation, and neglecting older electron techniques with uncertain doses, the risk estimates doubled compared to previous analyses to an excess odds ratio of 0.19 per Gy. Further scientific discussion is needed to clarify the consequences of this work on the assessment of radiation-induced risk of heart diseases. Additional insight might be gained by re-analyzing the Swedish part of the data set as well.

Radiation risk from radiotherapy applications can vary substantially between individuals. First, the doses do not only depend on the technique, but also vary with individual anatomy. For tangential breast cancer techniques, anatomic parameters have been identified that correlate with the doses and, consequently, the risks. It was shown that maximum heart distance, central lung distance, and minimum breast distance can explain the major part of individual variability in doses to the heart, lung and breast, respectively (Kong et al. 2002; Kundrát et al. 2019, 2022). Second, personal risk factors play an important role for long-term risk. Besides the high relevance of smoking for lung cancer risk (Cahoon et al. 2017), it was recently shown for women exposed at infancy for hemangioma that familial breast cancer history increases the risk of radiation-induced breast cancer almost threefold compared to women without familial breast cancer history (Eidemüller et al. 2021). Heart disease risk depends strongly on several risk factors, including a high cholesterol level, smoking or hypertension. In Darby et al. (2013) it was shown that radiation exposure seems to act multiplicatively with cardiac baseline risk. Thus, radiation risk can be substantially higher for patients with cardiac risk factors (Brenner et al. 2014; Simonetto et al. 2019b).

In summary, despite large efforts to estimate long-term health risks after breast cancer radiotherapy, risk assessment for modern techniques remains a big challenge. An important reason is the inhomogeneous exposure of organs close to the treatment area. To construct a dose–response relationship for the whole relevant dose range, a better mechanistic understanding of the biological processes underlying cell killing and repopulation and its consequences for radiation risk is needed. Furthermore, individual variability from doses and personal risk factors must be taken into account. This will reduce the current uncertainties related to risk assessment of second primary cancer and heart disease, and will help to optimize treatment regarding minimization of long-term risks.

## The immune system as main player in systemic radiation effects

Ionizing radiation induced effects on the immune system are largely dependent on the delivered dose and exposure scenario.

High dose total body exposure is a rare exposure scenario, which occurs as a result of an accidental overexposure (eg. nuclear accidents such as Chernobyl) or as a deliberate action (eg. A-bomb survivors). The major immune outcome is immune system failure, manifested as lymphocytopenia and granulocytopenia, which along with thrombocytopenia are the main causes of the hematopoietic syndrome in acute radiation sickness (DiCarlo et al. 2011).

High dose local exposure is typical in cancer treatment, where high doses of several tens of Gy are applied in a fractionated manner strictly to the tumor mass with a great care to spare surrounding healthy tissues as much as possible. Immune consequences of high dose irradiation within the tumor and its microenvironment are more complex and develop along different mechanisms compared to total body irradiation. Clinical observations and in vivo animal experiments show that local radiotherapy induces a selective rearrangement of tumor infiltrating lymphocytes by depleting radiosensitive immune cells such as CD8+ cytotoxic T cells and increasing the fraction of regulatory T cells and tumor infiltrating macrophages with an immune suppressive phenotype or by inhibiting CD8+ T cell responses in the tumor microenvironment through tumor infiltrating myeloid suppressor cells (Zhang et al. 2021; Shi et al. 2018).

In parallel, local radiotherapy, by inducing immunogenic cell death accompanied by increased release of danger signals activates pro-inflammatory mechanisms as well with immune stimulating outcome (Zhou et al. 2021; Frey et al. 2015; Baxevanis et al. 2022; Brandmaier and Formenti 2020; Zhu et al. 2021). Danger signals can activate professional antigen presenting cells, such as dendritic cells (DCs) (Lumniczky and Sáfrány 2015). It has been shown that in vivo irradiation of mice with 2 Gy leads to DC activation and a preference for DC interaction with effector CD4+ cells rather than Tregs, which again favors immune stimulation (Persa et al. 2018). Thus, local tumor irradiation leads to the activation of both immune stimulating and immune suppressing processes with a delicate balance between them (Lin et al. 2021). So far, no clear understanding exists of the factors which actually are able to shift this balance towards immune stimulation. Radiotherapy-induced abscopal effects, when local irradiation of a tumor leads to the regression of a distant metastasis, can be considered as distant out-of-field effects indicating that local radiotherapy has systemic consequences. Very little is known on how radiation-induced local immune activation becomes systemic. Recent data have proven that abscopal effects are mostly immune-mediated (Walle et al. 2018). Increasing evidence indicates a direct link between radiation-induced direct DNA damage, activation of the DNA damage response pathway and immune activation (Taffoni et al. 2021; Craig et al. 2021; Storozynsky et al. 2020). A better understanding of these interactions is needed to be able to quantify radiation-induced immune reactions and to characterize dose–response of complex immune reactions after radiation exposure. Based on local and systemic immune modulatory effects of radiotherapy currently great efforts are invested in developing immunotherapies which synergize with radiotherapy, clearly by enhancing irradiation-induced immune activation acting both locally on the primary tumor and systemically on distant metastasis (Haikerwal et al. 2015; Gandhi et al. 2015).

High dose irradiation of the tumors is inevitably accompanied by radiation exposure of the surrounding healthy tissues, the so-called out-of-field effects, detailed above, which can lead to radiotherapy-induced toxic side effects. Immune and inflammatory mechanisms are major drivers in the development of several of these toxic effects. Radiotherapy-induced pneumonitis and lung fibrosis is a much-feared side effect of radiotherapy delivered to tumors situated in the thorax region, where the pathological events are initiated by radiation-induced oxidative damage and tissue hypoxia and evolve in multiple waves. Induction of genes involved in inflammatory pathways, oxidative stress response, proliferation and angiogenesis leads to blood vessel dilatation, endothelial cell activation, infiltration of immune cells, release of inflammatory cytokines and development of an acute inflammation. If this is not resolved in time, oxidative stress persists and due to a positive feed-back a vicious circle develops, which maintains a chronic inflammatory status leading to progressive and irreversible fibrosis (Sprung et al. 2015; Guo et al. 2020; Käsmann et al. 2020). The mechanisms of radiotherapy-induced lung damage are extensively studied with the aim of finding reliable markers predicting individual sensitivity to radiotherapy-induced late tissue toxicity and with the scope of developing targeted therapies to mitigate radiation effects.

In contrast to high doses low dose local exposures of below 1 Gy have anti-inflammatory effects in individuals with local inflammatory conditions. This is used for treating patients with rheumatoid diseases with multiple fractions of low dose radiotherapy with the aim of reducing local inflammation and relieving pain. Moreover, similar anti-inflammatory effects are attributed to radon spa treatments as well, where patients are treated with radon doses mimicking a low dose total-body exposure equivalent with annual average background radon levels delivered in short treatment sessions. While the exact immune mechanisms responsible for this anti-inflammatory effect are by far not elucidated, it seems that changes in the balance of pro- and anti-inflammatory cytokines (most importantly transforming growth factor beta or TGF-β and tumor necrosis factor alpha or TNF-α) as well as enrichment of immune suppressive Tregs along with a reduction in effector T cell activation play important roles in this process (Rödel et al. 2012; Maier et al. 2020).

Immunological changes after acute or chronic low dose exposures can be the result of targeted effects, developing in cells directly hit by radiation or non-targeted effects manifesting in cells not directly hit by radiation. Based on epidemiological observations and experimental studies immunological effects developing after low dose exposure have certain characteristics: (a) effects are mild, and show high inter-individual variability; often changes are only evident compared to pre-exposure values in the same individual; (b) functional alterations prevail; (c) changes are persistent with slow and often incomplete regeneration; (d) low dose-induced effects mirror natural immune aging; (e) immune alterations indicate rather a perturbation of immune homeostasis or altered immune fitness and can rarely be directly related to certain diseases but data point to an increased predisposition to age-related chronic diseases of degenerative nature or cancer (Lumniczky et al. 2021).

## Out-of-field effects: model systems and mechanisms

Soluble factors, such as cytokines, chemokines and extracellular vesicles have been identified as systemic mediators of communication between immune cells and various other irradiated and non-irradiated tissues.

### Out-of-field radiation effects in the brain: proof of principle in rodent models

The contribution of systemic “out-of-field” effects to the risks of a long-term health detriment of radiation, especially in the brain, is still largely unknown. Indeed, both protective and damaging effects have been described (Mancuso et al. 2012), and no information on a dose–response relationship exists.

Pioneer work with the *Patched1* heterozygous knock out mice *(Ptch1*^+/–^), a well-characterized tumor mouse model in which ionizing radiation exposure dramatically accelerates tumor development in the brain and skin (Pazzaglia et al. 2002; Mancuso et al. 2004), has provided examples of in vivo out-of-field oncogenic radiation responses in the mouse brain. Cancer development in cerebellum of *Ptch1*^+/–^ mice was increased by radiation exposure of distant tissues, indicating that there is a level of communication between irradiated and non-irradiated tissues and organs (Mancuso et al. 2008). Noteworthy, decrease of tissue communication by ablation of one copy of *Connexin 43* (*Cx43*) gene, reduced the bystander tumor response in the cerebellum of *Ptch1*^+/–^ mice (Mancuso et al. 2011).

The effects of partial body exposure to ionizing radiation on brain cognitive functions are not clearly established in humans, and experimental data are scarce. However, several studies, reported hereafter provide in vivo examples of the existence of radiation-induced bystander non-cancer effects in the brain using rodent models. Investigations in rats showed that 6 h after partial-body exposure (head-protected) with 15 Gy of ^60^Co *γ*-rays, the brains had increased IL‐1β levels in the hypothalamus, thalamus and hippocampus, and increased levels of TNFα and IL-6 in the hypothalamus, suggesting that even body-only exposure, partial irradiation sparing the head, may produce oxidative stress and neuroinflammation in the brain (Marquette et al. 2003). Noteworthy, these responses were mediated by the vagus nerve and, were prevented by vagotomy before irradiation. In another study, examining the impact on the brain of non-brain directed radiation therapy, in which the mice were irradiated with 16 Gy of X-rays using a lead shield leaving only the right hind limb exposed, a global brain glucose hypometabolism, as well as acute and persistent multifocal microglial and astrocytic neuroinflammation, were reported in exposed mice (Feiock et al. 2016). Brain bystander effects after low-dose liver irradiation with 24.5 cGy of X-rays, manifested as altered gene and protein expression and DNA damage associated with neuroanatomical and behavioral changes, have also been reported in rats (Kovalchuk et al. 2016; Kovalchuk and Kolb 2017). Finally, altered brain morphology after focal irradiation of infant mice with X-rays (8 Gy), specifically targeting white matter (anterior commissure), neuronal (olfactory bulbs), or neurogenic (subventricular zone) regions, revealed that radiation damage locally can have important off-target consequences for brain development (Beera et al. 2018).

Results of a series of experiments involving exposure with heavy particles which vary linear energy transfer (LET), related to space travel, sometimes produced differing results. Changes in neuronal function and cognitive performance could be observed following both head-only (body shielded with overlapping tungsten bricks of 10 cm) and whole-body exposures to ^4^He particles (1000 MeV/n, LET ≈ 0.9 keV/μm, 0.01–0.50 cGy) in rats at doses as low as 0.01–0.025 cGy (Rabin et al. 2019). However, results from another study using ^16^O particles reported that whole-body exposure was more effective in disrupting cognitive performance than head-only exposure, in that whole-body exposure affected performance at a lower dose than head-only exposure (25 compared to 50 cGy) in the initial test of performance 3 months following exposure (Rabin et al. 2014), suggesting that body exposure affect cognition. In addition, recent investigations on the effects that body irradiation might have on neuronal function and cognitive performance, in which rats were given head-only, body-only or whole-body exposures to 25 or 50 cGy of ^56^Fe particles (600 MeV/n), showed that body-only exposure is capable of affecting cognitive performance, suggesting that disruption of neuronal function and cognitive performance after exposure to HZE particles is not dependent upon the direct effect on neurons (Cahoon et al. 2020) and that hits from HZE particles anywhere on the body concur to risk to the central nervous system (CNS). However, despite these reports demonstrating physiological consequences of ionizing radiation bystander effects on the CNS, a global understanding of the out-of-field brain radiation-induced effects, especially within the context of an intact mammalian organism, is still lacking.

Recently, in the EURATOM funded project “SEPARATE” (Systemic Effect of Partial-body Exposure to Low Radiation Doses, 2018–2020), a multiomic approach was adopted to investigate in vivo out-of-field non-cancer responses in several organs, including the hippocampus, following partial irradiation. Mice irradiated with low/moderate radiation doses (0.1 Gy/2 Gy) in the lower third of the body with the upper two third shielded, displayed changes in non-coding RNAs, proteins and metabolic levels in the hippocampus, as well as defects in neurogenesis very similar to those induced by whole-body exposures, providing a proof of principle of the existence of out-of-field radiation response in the hippocampus (Pazzaglia et al. 2021). In vivo investigations on radiation responses in mice, allow to unravel the mechanistic features of targeted and non-targeted radiation responses in the hippocampus, also providing a greater understanding of radiation-induced bystander effect and of its clinical implications in the pathogenesis of radiation-induced neurocognitive dysfunction. Since this might have important implications, including radiation therapy, further investigations to disentangle direct and out-of-field effects deriving from radiation exposure are recommended.

### Ionizing radiation induced cardiovascular effects

Adjuvant radiotherapy is an effective treatment for thoracic malignancies, however, incidental radiation exposure to the heart and large arteries during treatment is unavoidable, potentially resulting in secondary cardiovascular disease (CVD), especially atherosclerosis, in cancer survivors (Stewart et al. 2012; Darby et al. 2013; Little et al. 2016; Darby et al. 2010). Atherosclerosis is a progressive inflammatory disease and the pathogenesis involves a complex interplay of local inflammation which is associated with increased expression of pro-inflammatory cytokines, chemokines and adhesion molecules, oxidative stress, cell death, lipid accumulation, and smooth muscle cell proliferation, resulting in the formation of atherosclerotic plaques. The underlying pathophysiology of radiation-induced CVD atherosclerosis is complex and the mechanisms remain incompletely understood, possibly resulting in improper radiation protection.

**Fig. 1:**
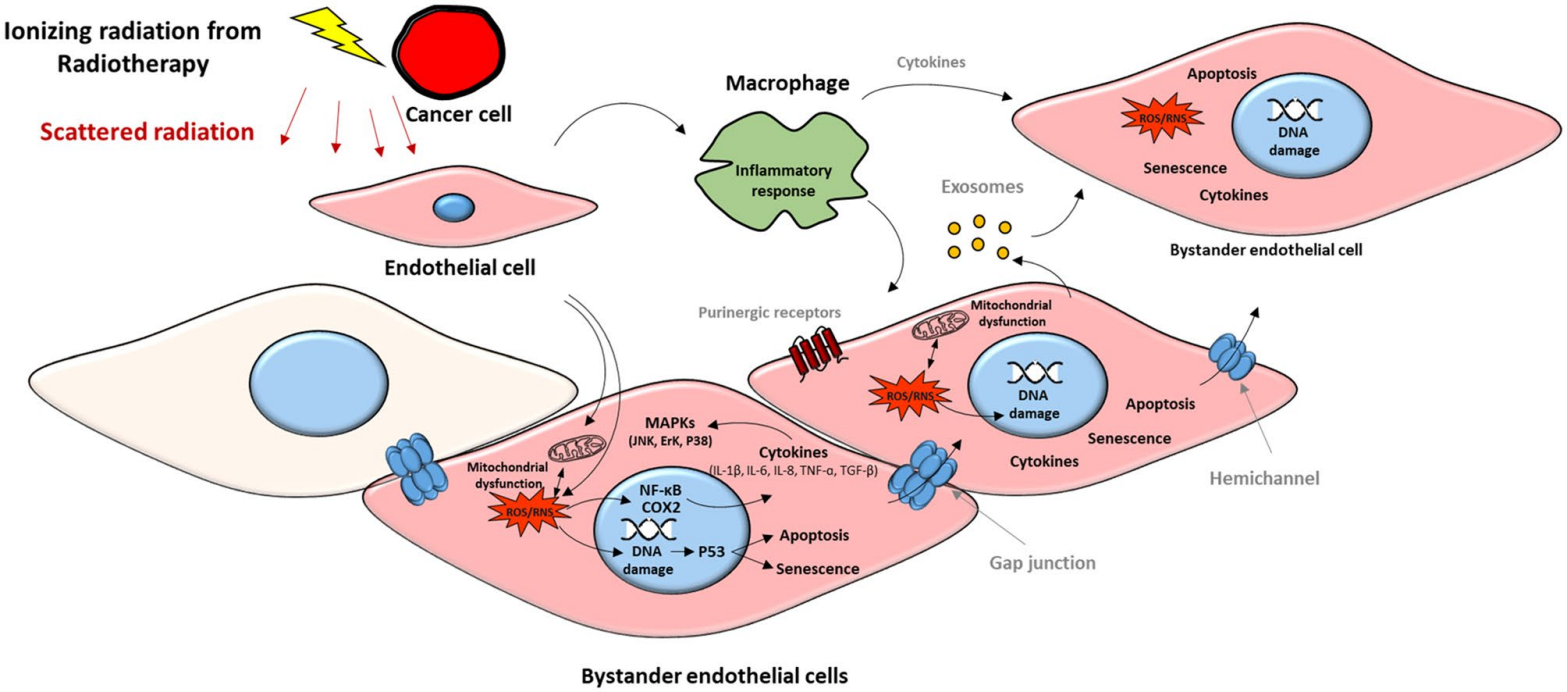
Hypothetical mechanism of action of radiation-induced bystander endothelial dysfunction. During radiotherapy of thoracic cancer, endothelial cells may receive scattered irradiation and may produce bystander responses to non-irradiated endothelial cells in the cardiovascular system via two main routes: (i) by direct cell-to-cell communication mediated by gap junctions, (ii) by extracellular vesicles (e.g. exosomes) and (iii) by paracrine release of soluble factors such as ATP, released via vesicular mechanisms or hemichannels to the extracellular environment. Macrophages might be important mediators by regulating inflammatory response and cytokine release to bystander cells. Reactive oxygen and nitrogen species (ROS/RNS), signal transduction through p53, MAPKs and NF-κb together with signaling cyclooxygenase-2 (COX2) may be involved in bystander responses in non-targeted endothelial cells after ionizing radiation exposure. Eventually, these signaling molecules may play a role in endothelial cell dysfunction by triggering DNA damage, mitochondrial dysfunction, inflammatory responses, apoptosis and senescence. Not all the cells are affected by bystander signaling (yellow cell). Figure adapted from (Ramadan et al. 2021a, b)

The initiating step of atherosclerosis is the damage to the vascular endothelial cells, therefore, endothelial cells are reasonable in vitro model to study the effect of radiation exposure on the cardiovascular system, especially in terms of defining early initiating events. It was hypothesized that radiation exposure may cause macrovascular as well as microvascular damage, which may act together to produce coronary artery disease after radiotherapy (Darby et al. 2010). This macrovascular and microvascular damage could be triggered during thoracic radiotherapy by the scattered radiation doses received in the heart region, which may damage the endothelium directly, by inducing DNA damage, oxidative stress, cell death, inflammation, and premature cell senescence to initiate the atherosclerosis process, that may expand via bystander signaling to non-irradiated endothelial cells as shown in Fig. 1 (Ramadan et al. 2021a, b).

Although radiation-induced bystander effect (RIBE) has improved our understanding of the non-targeted effects after radiotherapy, RIBE in the development of radiation-induced atherosclerosis is still poorly defined. Two main routes were reported to regulate bystander signals: (i) direct cell-to-cell communication, often mediated by gap junction (GJ) communication and (ii) paracrine release of soluble factors from directly irradiated cells to the extracellular environment, which could be mediated by exosomes release and purinergic signaling through the P2X family or plasma membrane hemichannels (HC) (Little 2006; Ohshima et al. 2012; Xu et al. 2015; Tsukimoto et al. 2010). GJs and HC are composed of a transmembrane protein called connexin (Cx). Endothelial Cxs play an important role in atherosclerosis development. Cx37 and Cx40 are almost absent in the endothelium covering the advanced atherosclerotic plaques in mouse and human; however, they are normally distributed in the endothelium of healthy arteries. While Cx43 is highly expressed at specific regions of advanced atherosclerotic plaques (Kwak et al. 2002). It was reported by our group that IR induces a dose-dependent upregulation of proatherogenic Cx43 and downregulation of atheroprotective Cx40 gene and protein levels in human immortalized coronary artery and microvascular endothelial cells (Ramadan et al. 2019). In addition, IR increases GJ intercellular communication and induces acute and long-lived Cx43 HC opening in a dose-dependent manner in these endothelial cells (Ramadan et al. 2019). Excessive Cx43 HC opening is considered a pathological condition, since it results in ATP leakage that acts in a paracrine manner on surrounding cells, and can activate downstream cellular processes including oxidative stress responses, apoptosis, propagating intercellular Ca2+ waves, NLRP3 inflammasome pathway activation and inflammation (Decrock et al. 2009a, b; 2017; Hoorelbeke et al. 2020), which are known to be involved in the pathogenesis of radiation-induced atherosclerosis (Huang et al. 2020; Wijerathne et al. 2021). Moreover, IR induced an increase in GJ coupling and Cx43 HC opening may act to spread radiation damaging responses to neighboring non-irradiated cells, possibly amplifying endothelial cell damage (Hoorelbeke et al. 2020; Autsavapromporn et al. 2011; Decrock et al. 2009a, b). We further showed that Cx43 HC contribute to radiation-induced coronary artery and microvascular endothelium damage in vitro by mediating oxidative stress, cell death, premature cell senescence and pro-inflammatory and pathological factors like the Pro-Inflammatory Cytokines IL-1β and IL-8, the adhesion molecule VCAM-1, the chemokine MCP-1 and the vasoconstrictor peptide endothelin-1 (Ramadan et al. 2020), which was linked to endothelial cell dysfunction by reducing nitric oxide (NO) vasodilatory signalling (Bohm et al. 2007). These events could be protected by Cx43 HC-inhibiting peptide TAT-Gap19. Therefore, targeting Cx43 HC may hold potential to protect against radiation-induced endothelial cell damage. Although endothelial cell models in these in vitro experiments provided an insight on the role of intercellular communication after radiation exposure and helped to understand the mechanisms at a single layer of cells, they are not completely representative to the physiological in vivo complex situation which involves different layers of responses including the immune response, therefore, validation studies in vivo models are warranted.

Although cardiovascular complications often occur 10–15 years after radiation exposure, in the form of accelerated atherosclerosis, early asymptomatic changes in the cardiovascular system may occur in more direct relation to irradiation, before the appearance of disease symptoms. Therefore, pre-clinical investigations on early cardiovascular responses may improve our knowledge on asymptomatic changes in the cardiovascular system after IR exposure and may help in early detection of patients at risk for developing CVD after radiotherapy. For that purpose, our group has investigated the acute and early term changes in the cardiovascular system after local thoracic irradiation of atherosclerotic prone ApoE-/- mice by investigating systemic cholesterol and triglycerides levels, as well as a large panel of inflammatory markers at 24 h and 1 month after exposure. Wild type mice models are resistant to atherosclerosis development due to the low level of low-density lipoprotein (LDL), therefore, apolipoprotein E-deficient (ApoE−/−) mice and LDL receptor knock-out mice which display poor lipoprotein clearance with subsequent accumulation of cholesterol, that promote the development of atherosclerotic plaques, are the most common mice models to study the pathophysiology of atherosclerosis. We observed increased serum GDF-15 and CXCL10 in both female and male ApoE−/− mice at 24 h after low and high dose local thoracic irradiation, which was validated to be secreted from the coronary artery and microvascular endothelial cells in vitro (Ramadan et al. 2021a, b). GDF-15 and CXCL10 are proatherogenic inflammatory markers, and they are promising biomarkers in cardiovascular diseases in humans, including atherosclerosis (Xu et al. 2011; Tavakolian Ferdousie et al. 2017). GDF-15 is a member of the transforming growth factor β superfamily that may contribute to the initiation and the progression of atherosclerotic lesions by regulating cell death and IL-6–dependent inflammatory responses (Bonaterra et al. 2012), and by contributing to plaque instability (De Jager et al. 2011). CXCL10 acts as a chemoattractant cytokine and was shown to promote atherosclerosis by recruitment and retention of activated T lymphocytes to vascular wall lesions during the atherosclerotic process (Heller et al. 2006). Further research is needed to assess GDF-15 and CXCL10 levels in radiotherapy-treated patients and to explore the possibility of using them as a potential biomarker to early detect the risk of cardiovascular diseases in the thoracic radiotherapy-treated patient, thus identifying patients who may benefit from early medical intervention.

In addition to the correlation between therapeutic radiation doses and cardio-vascular risks, epidemiological evidence has established a link between cardiovascular disease and exposure of the heart and major vessels to doses above 500 mGy (Schultz-Hector et al. 2007; Hendry et al. 2008; Little et al. 2008). Nevertheless, at lower doses the evidence for a detrimental effect is inconclusive due to the lack of appropriate epidemiological studies, coupled with lack of knowledge of the processes involved that is needed for construction of mathematical models. In this context, an acute high dose of 6 Gy and a moderate 0.3 Gy dose were reported to have a significant impact on development of atherogenesis in a predisposed mouse model (ApoE-/- mice), although with different mechanisms, i.e., predominantly lesion formation at high doses and growth of existing lesions at low doses (Mancuso et al. 2015). These results suggest that lower doses, such as those typically received in the nuclear workplace or from diagnostic examinations such as CT scanning, may be more damaging than predicted by a linear dose response and open new questions on the potential abscopal actions of radiation on the cardiovascular system.

Furthermore, within the “SEPARATE” project, investigations on in vivo out-of-field effects in the heart, following partial body irradiation (PBI) have also been addressed. Through a miRNome NGS-based analysis we identified changes in miRNAs in out-of-field heart 15 days after PBI with 2 Gy of X-rays that were also detected in corresponding tissue of whole body irradiated (WBI) mice, demonstrating the existence of out-of-field radiation response also in the heart of conventional mice. In addition, at longer post-irradiation time (6 months), both WBI and PBI hearts showed a clear upregulation of miRNAs known as master regulators of fibrosis and over half of the miRNAs deregulated in the heart (54%) was also deregulated in out-of-field hippocampus. These findings suggested that even PBI, through molecular mechanisms mediated by miRNAs and partially overlapping with those acting after WBI, have the potential to induce reactions in multiple shielded tissue (Manuscript in preparation).

### Extracellular vesicles (EVs) as mediators of out-of-field radiation effects

Current literature, including examples presented during this workshop convincingly indicate systemic radiation effects in non-targeted tissues. However, mechanisms and transmitters of such radiation effects are less clear. It is well established that irradiated cells release a variety of soluble factors, which are either released to the extracellular space or exchanged between cells through gap junctions or tunnelling nanotubes. These factors can be single molecules such as chemokines, cytokines and reactive oxygen species, due to the diversity of effects also multiple coordinated signals are suggested (Hei et al. 2008; Morgan and Sowa 2015). As an attractive mediator of such multiple signals during radiation response extracellular vesicles (EVs) were intensively discussed during the workshop. The role of EVs as an emerging topic is stressed by around 500 Pubmed registered publications covering the search query (extracellular vesicles or exosomes and radiation) within the last two years. Furthermore, two recently EU-funded projects, LEU-TRACK and SEPARATE addressed the function of EVs in radiation response.

EVs are a heterogeneous group of phospholipid-membrane surrounded vesicles, which are released from almost all cell types (Niel et al. 2018). The EV cargo includes mRNA, small and long non-coding RNAs, genomic DNA fragments, proteins, metabolites and lipids. Either by acting as ligands for cell surface structures or by internalization leading to the release of their cargo into recipient cells, EVs play important roles in cell communication, both locally and systemically (Szatmári et al. 2017; Kadhim et al. 2022). Traditionally EVs are divided into different subgroups depending on their biogenesis and size after release. Exosomes are the smallest subtype of EVs (50–150 nm). They are generated as an inward budding of the membranes of early endosomes. Microvesicles (100–1000 nm) are a result of direct outward blebbing of the plasma membrane into the extracellular space. Apoptotic bodies (100–5000 nm) are a class of EVs that are generated during the final phase of apoptosis by a process called "Apoptotic blebbing". In size, they are the largest types of EVs. In comparison with other types of EVs, apoptotic bodies are less known for being 'safe containers' of EV cargo, as they are phagocytosed and degraded shortly after their release into the extracellular space. According to the International Society of Extracellular Vesicles (ISEV), extracellular vesicles are divided either on their size, their biochemical composition or on the cells of origin. In practice, most often categorization is made into small EVs (below 200 nm) and large EVs (above 200 nm) based on vesicle size (Théry et al. 2018).

Radiation interferes with EV biogenesis, release and destination on several levels and may contribute to systemic radiation effects. The content of EVs, primarily, depends on the type and the state of the donor cell, but stress conditions, including radiation, affect EV release and composition. Moreover, radiation may affect EV release as well as their interaction with recipient cells.

It has been shown that whole body irradiation influences the secretion of EVs from bone marrow cell subpopulations in mice: EVs containing mesenchymal stem cell markers CD29 and CD44 decreased, while EVs with haematopoietic stem cell or lymphoid progenitor markers increased after irradiation (Kis et al. 2020). Several studies suggest increased EV release after irradiation in in vitro and in vivo models. For example, elevated EV release after irradiation with therapeutically relevant doses was evidenced in tumor cell culture models, such as head and neck cancer and glioblastoma (Mutschelknaus et al. 2017; Arscott et al. 2013). After partial body irradiation a recent study showed increased EV contents in the brain, heart and liver of mice (Tunkay-Cagatay et al. 2020). As potential mechanism for the radiation increased EV release, p53-mediated pathways are suggested (Yu et al. 2006).

Numerous studies describe radiation-induced changes in the EV composition. The identified changes are diverse and there is no common signature for radiation exposure identified in EVs, yet. Alterations seem to be highly related to cell type, radiation dose and also time post exposure. This refers especially to tumour cells, where a plethora of microRNA and protein changes were described. However, a growing body of evidence indicates that non-cancer cells, such as peripheral blood mononuclear cells (ex vivo irradiated) or bone marrow cells (in vivo, total body) (Moertl et al. 2020; Beer et al. 2015; Szatmári et al. 2017) are capable of releasing EVs with changed compositions. Radiation induced EV alterations after partial body irradiation seem to be especially important for systemic radiation effects. A study by Hinzman et al. showed acute and chronic changes in plasma-derived EV after cranial irradiation of mice. By metabolomic and lipidomic profiling they found an enrichment of factors involved in inflammation that may mediate systemic response to distant organ sites (Hinzman et al. 2019). Using Raman spectroscopy radiation induced alterations in protein and nucleic acid features were suggested 24 h as well as 15d post partial body irradiation in a mouse model (Tunkay-Cagatay et al. 2020). Also, tumor cell irradiation in radiotherapy patients induced EV cargo changes at distinct sites. For example, differential expression of serum exosomal miRNAs is monitored in prostate cancer or glioma patients after radiotherapy, which may have potential value as prognostic and predictive biomarkers (Malla et al. 2018; Li et al. 2021).

There is growing evidence that EVs are a substantial, functional component of the cellular radiation response. Recent data suggest a complex network of interactions between EVs from irradiated cancer and non-cancer cells with cancer and non-cancer recipient cells, which contribute to systemic radiation effects in irradiated and non-irradiated areas. Many in vitro functional studies focus on EVs in the tumour radiation response. Increasing evidence suggests that EVs play a significant role in facilitating the development of radioresistance and motility in cancer cells. In glioblastoma cell culture models, Mrowczynski et al. (2018) discovered that exosomes enhance cell survival after radiation exposure by increasing levels of oncogenic miRNAs, mRNAs and pro-survival pathway proteins and at the same time decreasing levels of tumor-suppressive miRNAs and mRNAs. In the same cancer type a promigratory role of radiation-related EVs was reported (Arscot et al. 2013). Likewise, EVs from irradiated head and neck cancer and neuroblastoma cells stimulate survival, migration and invasiveness in in vitro approaches (Mutschelknaus et al. 2016, 2017; Tortolici et al. 2021). However, there are also studies reporting the induction of harmful effects of EVs from irradiated cancer cells in recipient cells, like increased chromosomal damage and increased ROS levels induced by EVs from irradiated MCF-7 breast cancer cells (Al-Mayah et al. 2012; Nakaoka et al. 2021). Tumour-derived extracellular vesicles act not only on themselves, but also on stromal cells, like dendritic and endothelial cells. Upon irradiation, EVs derived from gastric cancer and lung cancer cell culture cells promote the proliferation, migration, and invasion of endothelial cells (Li et al. 2018; Zheng et al. 2017). Given that EVs are secreted by almost all cell types, also EVs derived from stromal cells are suggested to influence cancer cells and other types of stromal cells in the context of radiation exposure (reviewed in He et al. 2021). Beside tumour- and tumour associated cells, normal cells communicate radiation signals through EVs. Early work by Jella et al. showed the transmission of cytotoxic effects between irradiated and non- irradiated keratinocytes in an in vitro model system (Jella et al. 2014). EVs from the bone marrow of whole body irradiated mice induced a redistribution in the bone marrow cell subpopulations, oxidative and chromosomal damage, altered the antioxidant system and elicited immunological changes in non-irradiated recipient mice, which basically resembled the effects induced by direct radiation (Szatmari et al. 2017, 2019; Hargitai et al. 2021). Many of these effects were present for a minimum of three months after EV injection if EVs were isolated from acutely irradiated mice but bystander effects resolved if EVs originated from a bone marrow irradiated three months earlier (Kis et al. 2022). After partial body irradiation changed EV characteristics were reported in distant non-irradiated organs, which were able to induce phenotypic changes in co-cultivated MEFs (Tuncay-Cagatay et al. 2020). On the other hand, several reports found beneficial effects, such as pro-survival or pro-proliferative effects, of EVs released from ex vivo irradiated human PBMCs. In this regard, EVs from irradiated blood cells were shown to reduce radiation induced apoptosis in endothelial cells (Moertl et al. 2020). Accordingly, pro-angiogenic and tissue regenerative capacities were attributed to EVs from ex vivo irradiated PBMC (Wagner et al. 2018; Beer et al. 2015). Finally, circulating exosomes derived from plasma of partial-body- and whole-body-irradiated mice, exhibit changes in both miRNA and protein cargo compared to those from unexposed mice. When intracranially injected in the neonatal mouse cerebellum, exosomes from irradiated mice were shown to attenuate neuro-inflammatory response and protect from apoptosis in vivo (Pazzaglia et al. 2022), holding promise for exosome-based future therapeutic applications against radiation injury.

The available observations indicate a vital role of EVs in the radiation response of cancer and non-cancer cells. Radiation affects not only the production and the composition of EVs but also their phenotypes in recipient cells. Therefore, these mechanisms can contribute to the systemic distribution of local radiation effects throughout an organism. Hence, understanding EV-mediated communication during radiation response is critical for a better understanding of radiation induced health effects with importance for radiotherapy and radiation protection. In the future EVs may be used as biomarkers for radiation exposure and predictors of radiation effects. In the context of radiotherapy EVs are attractive targets to improve therapy efficiency by artificially engineering EV surface and cargo to achieve a selective targeting as well as an improved tumor radiosensitization (Szatmári et al. 2019). However, in advance of such applications, there are still many challenges for EV studies in the radiation field. Major points are the elucidation of the dynamics of IR-induced alternation in EV secretion, composition and function according to dose and temporal effects and the investigation of EV subpopulations together with cell-type specific functions.

## Conclusions and future directions

The presentations and discussions at the workshop highlighted the relevance of partial body exposures and radiation-induced out-of-field effects. Partial body exposures and spatial dose variations occur frequently in the real world and are the norm for exposures in radiation therapy, diagnostic radiology and in occupational settings. At the same time, such exposure conditions pose big scientific challenges for the evaluation of health risks. The biological mechanisms and clinical relevance of out-of-field effects are still poorly understood. Furthermore, assessment of doses and risks for radiotherapy-related cancer and non-cancer diseases becomes increasingly relevant with new treatment modalities and improved life expectancy.

In summary, systemic radiation effects in non-targeted tissues have been experimentally demonstrated for different organs in animal model systems, including bone marrow, brain, cardiovascular and immune system. Radiation-induced alterations in the pattern of expression of connexins and miRNAs, for instance, have been identified in the cardiovascular system that may potentially mediate out-of-field reactions. Great efforts are also being invested in elucidating the contribution EVs as a mediator of systemic radiation effects and radiation exposure has been shown to affect both EV release and cargo, by increasing their secretion and modulating the EV bioactive cargo. As far as future directions are concerned, understanding the health effects of inhomogeneous radiation exposures still remain a key priority for radiation protection research and for MELODI (https://melodi-online.eu/wp-content/uploads/2021/10/2021-MELODI-Statement-draft-FINAL-post-consultation.pdf). In general, knowledge of the mechanisms responsible for biological effects of inhomogeneous dose deposition both for cancer and non-cancer diseases is still limited and relevant experimental models or valid datasets are sparse. Therefore, suitable tissue and in vivo models for the quantification of the impact of dose inhomogeneity should be developed. In addition, the identification of relevant pathways in a systems biology approach might help to characterize the response of the complex system as a whole. Also, the use of the adverse outcome pathway (AOP) approach may provide help to identify relevant pathways in case of inhomogeneous exposures. Among other research gaps, it is important to identify high-risk organs in patients exposed to inhomogeneous fields, as well as development of early biomarkers. Finally, modeling may play an important role in bridging the information from clinical/epidemiology and mechanistic studies for a better risk assessment. In particular, developing new models of dose calculation for healthy tissues (especially for new treatment modalities, such as proton irradiation) and to improve risk models and personalize risk assessment for cancer and non-cancer effects from radiotherapy applications are also important priorities for future research.

## Footnotes

1 With regard to dose ranges in radiotherapy, we refer to doses above about 4 Gy as high doses, and to doses below about 4 Gy as low- and medium doses.

## References

[CR1] Al-Mayah AH, Irons SL, Pink RC, Carter DR, Kadhim MA (2012). Possible role of exosomes containing RNA in mediating nontargeted effect of ionizing radiation. Radiat Res.

[CR2] Arscott WT, Tandle AT, Zhao S, Shabason JE, Gordon IK, Schlaff CD, Zhang G, Tofilon PJ, Camphausen KA (2013). Ionizing radiation and glioblastoma exosomes: implications in tumor biology and cell migration. Transl Oncol.

[CR3] Autsavapromporn N, de Toledo SM, Little JB, Jay-Gerin JP, Harris AL, Azzam EI (2011). The role of gap junction communication and oxidative stress in the propagation of toxic effects among high-dose α-particle-irradiated human cells. Radiat Res.

[CR4] Baiocco G, Bartzsch S, Conte V, Friedrich T, Jakob B, Tartas A, Villagrasa C, Prise K (2022). A matter of space: how the spatial heterogeneity in energy deposition determines the biological outcome of radiation exposure. Radiat Environ Biophys.

[CR5] Baxevanis CN, Gritzapis AD, Voutsas IF, Batsaki P, Goulielmaki M, Adamaki M, Zoumpourlis V, Fortis SP (2022). T cell repertoire in tumor radiation: the emerging frontier as a radiotherapy biomarker. Cancers (basel).

[CR6] Beer L, Zimmermann M, Mitterbauer A, Ellinger A, Gruber F, Narzt MS, Zellner M, Gyöngyösi M, Madlener S, Simader E, Gabriel C, Mildner M, Ankersmit HJ (2015). Analysis of the secretome of apoptotic peripheral blood mononuclear cells: impact of released proteins and exosomes for tissue regeneration. Sci Rep.

[CR7] Beera KG, Li YQ, Dazai J, Stewart J, Egan S, Ahmed M, Wong CS, Jaffray DA, Nieman BJ (2018). Altered brain morphology after focal radiation reveals impact of off-target effects: implications for white matter development and neuro-genesis. Neuro Oncol.

[CR8] Belzile-Dugas E, Eisenberg MJ (2021). Radiation-induced cardiovascular disease: review of an underrecognized pathology. J Am Heart Assoc.

[CR9] Berkey FJ (2010). Managing the adverse effects of radiation therapy. Am Fam Physician.

[CR10] Berrington de Gonzalez AB, Gilbert E, Curtis R, Inskip P, Kleinerman R, Morton L, Rajaraman P, Little MP (2013). Second solid cancers after radiation therapy: a systematic review of the epidemiologic studies of the radiation dose-response relationship. Int J Radiat Oncol Biol Phys.

[CR11] Boei J, Fenske N, Hofmann W, Madas BG, Mezquita L (2022). Effects of spatial variation in dose delivery: what can we learn from radon induced lung cancer studies. Radiat Environ Biophys.

[CR12] Böhm F, Pernow J (2007). The importance of endothelin-1 for vascular dysfunction in cardiovascular disease. Cardiovasc Res.

[CR13] Bonaterra GA, Zügel S, Thogersen J, Walter SA, Haberkorn U, Strelau J, Kinscherf R (2012). Growth differentiation factor-15 deficiency inhibits atherosclerosis progression by regulating interleukin-6-dependent inflammatory response to vascular injury. J Am Heart Assoc.

[CR14] Bordy JM, Bessieres I, d'Agostino E, Domingo C, d'Errico F, di Fulvio A, Knezevic Ž, Miljanić S, Olko P, Ostrowsky A, Poumarede B (2013). Radiotherapy out-of-field dosimetry: Experimental and computational results for photons in a water tank. Rad Meas.

[CR15] Brandmaier A, Formenti SC (2020). The impact of radiation therapy on innate and adaptive tumor immunity. Semin Radiat Oncol.

[CR16] Brenner DJ, Shuryak I, Jozsef G, Dewyngaert KJ, Formenti SC (2014). Risk and risk reduction of major coronary events associated with contemporary breast radiotherapy. JAMA Intern Med.

[CR17] Cahan WG, Woodard HQ (1948). Sarcoma arising in irradiated bone; report of 11 cases. Cancer.

[CR18] Cahoon EK, Preston DL, Pierce DA, Grant E, Brenner AV, Mabuchi K, Utada M, Ozasa K (2017). Lung, laryngeal and other respiratory cancer incidence among Japanese atomic bomb survivors: an updated analysis from 1958 through 2009. Radiat Res.

[CR19] Cahoon DS, Shukitt-Hale B, Bielinski DF, Hawkins EM, Cacioppo AM, Rabin BM (2020). Effects of partial- or whole-body exposures to 56Fe particles on brain function and cognitive performance in rats. Life Sci Space Res (amst).

[CR20] Corradini S, Ballhausen H, Weingandt H, Freislederer P, Schönecker S, Niyazi M, Simonetto C, Eidemüller M, Ganswindt U, Belka C (2018). Left-sided breast cancer and risks of secondary lung cancer and ischemic heart disease: effects of modern radiotherapy techniques. Strahlenther Onkol.

[CR21] Craig DJ, Nanavaty NS, Devanaboyina M, Stanbery L, Hamouda D, Edelman G, Dworkin L, Nemunaitis JJ (2021). The abscopal effect of radiation therapy. Future Oncol.

[CR22] Darby SC, Cutter DJ, Boerma M, Constine LS, Fajardo LF, Kodama K (2010). Radiation-related heart disease: current knowledge and future prospects. Int J Radiat Oncol Biol Phys.

[CR23] Darby SC, Ewertz M, McGale P, Bennet AM, Blom-Goldman U, Bronnum D (2013). Risk of ischemic heart disease in women after radiotherapy for breast cancer. N Engl J Med.

[CR24] de Jager SC, Bermudez B, Bot I (2011). Growth differentiation factor 15 deficiency protects against atherosclerosis by attenuating CCR2-mediated macrophage chemotaxis. J Exp Med.

[CR25] De Saint-Hubert M, Verbeek N, Bäumer C, Esser J, Wulff J, Nabha R, Van Hoey O, Dabin J, Stuckmann F, Vasi F, Radonic S, Boissonnat G, Schneider U, Rodriguez M, Timmermann B, Thierry-Chef I, Brualla L (2022). Validation of a Monte Carlo framework for out-of-field dose calculations in proton therapy. Front Oncol.

[CR26] De Saint-Hubert M, Farah J, Klodowska M, Romero-Expósito MT, Tyminska K, Mares V (2022). The influence of nuclear models and Monte Carlo radiation transport codes on stray neutron dose estimations in proton therapy. Rad Meas.

[CR27] Decrock E, De Vuyst E, Vinken M, Van Moorhem M, Vranckx K, Wang N (2009). Connexin 43 hemichannels contribute to the propagation of apoptotic cell death in a rat C6 glioma cell model. Cell Death Differ.

[CR28] Decrock E, Vinken M, De Vuyst E, Krysko DV, D'Herde K, Vanhaecke T (2009). Connexin-related signaling in cell death: to live or let die. Cell Death Differ.

[CR29] Decrock E, Hoorelbeke D, Ramadan R, Delvaeye T, De Bock M, Wang N (2017). Calcium, oxidative stress and connexin channels, a harmonious orchestra directing the response to radiotherapy treatment. Biochim Biophys Acta.

[CR30] Di Fulvio A, Tana L, Caresana M, D'Agostino E, de San PM, Domingo C, d’Errico F (2013). Clinical simulations of prostate radiotherapy using BOMAB-like phantoms: results for neutrons. Rad Meas.

[CR31] Diallo I, Haddy N, Adjadj E, Samand A, Quiniou E, Chavaudra J, Alziar I, Perret N, Guerin S, Lefkopoulos D, de Vathaire F (2009). Frequency distribution of second solid cancer locations in relation to the irradiated volume among 115 patients treated for childhood cancer. Int J Radiat Oncol Biol Phys.

[CR32] DiCarlo LA, Maher C, Hick JL, Hanfling D, Dainiak N, Chao N, Bader JL, Coleman NC, Weinstock DM (2011). Radiation injury after a nuclear detonation: medical consequences and the need for scarce resources allocation. Disaster Med Public Health Prep.

[CR33] Ding GX, Alaei P, Curran B, Flynn R, Gossman M, Mackie TR, Miften M, Morin R, Xu XG, Zhu TC (2018). Image guidance doses delivered during radiotherapy: quantification, management, and reduction: report of the AAPM Therapy Physics Committee Task Group 180. Med Phys.

[CR34] Eidemüller M, Simonetto C, Kundrát P, Ulanowski A, Shemiakina E, Güthlin D, Rennau H, Remmele J, Hildebrandt G, Wolf U (2019). Long-term health risk after breast cancer radiotherapy: overview of PASSOS methodology and software. Radiat Prot Dosim.

[CR35] Eidemüller M, Holmberg E, Lundell M, Karlsson P (2021). Evidence for increased susceptibility to breast cancer from exposure to ionizing radiation due to a familial history of breast cancer: results from the Swedish Hemangioma Cohort. Am J Epidemiol.

[CR36] Feiock C, Yagi M, Maidman A, Rendahl A, Hui S, Seelig D (2016). Central nervous system injury—a newly observed bystander effect of radiation. PLoS ONE.

[CR200] Formenti SC, Demaria S (2009) Systemic effects of local radiotherapy. The Lancet Oncol 10(7):718–726. 10.1016/S1470-2045(09)70082-8 10.1016/S1470-2045(09)70082-8 PMC2782943 19573801

[CR37] Frey B, Hehlgans S, Rödel F, Gaipl US (2015). Modulation of inflammation by low and high doses of ionizing radiation: Implications for benign and malign diseases. Cancer Lett.

[CR38] Gandhi SJ, Minn AJ, Vonderheide RH, Wherry EJ, Hahn SM, Maity A (2015). Awakening the immune system with radiation: optimal dose and fractionation. Cancer Lett.

[CR39] Grant EJ, Brenner A, Sugiyama H, Sakata R, Sadakane A, Utada M, Cahoon EK, Milder CM, Soda M, Cullings HM, Preston DL, Mabuchi K, Ozasa K (2017). Solid cancer incidence among the Life Span Study of atomic bomb survivors: 1958–2009. Radiat Res.

[CR40] Guo T, Zou L, Ni J, Zhou Y, Ye L, Yang X, Zhu Z (2020). Regulatory T cells: an emerging player in radiation-induced lung injury. Front Immunol.

[CR41] Haikerwal SJ, Hagekyriakou J, MacManus M, Martin OA, Haynes NM (2015). Building immunity to cancer with radiation therapy. Cancer Let.

[CR42] Halg RA, Schneider U (2020). Neutron dose and its measurement in proton therapy-current state of knowledge. Br J Radiol.

[CR43] Halg RA, Besserer J, Schneider U (2012). Systematic measurements of whole-body imaging dose distributions in image-guided radiation therapy. Med Phys.

[CR44] Hargitai R, Kis D, Persa E, Szatmári T, Sáfrány G, Lumniczky K (2021). Oxidative stress and gene expression modifications mediated by extracellular vesicles: an in vivo study of the radiation-induced bystander effect. Antioxidants.

[CR45] Harrison R (2017). Out-of-field doses in radiotherapy: input to epidemiological studies and dose-risk models. Phys Med.

[CR46] Hauri P, Schneider U (2019). Whole-body dose equivalent including neutrons is similar for 6 MV and 15 MV IMRT, VMAT, and 3D conformal radiotherapy. J Appl Clin Med Phys.

[CR47] He C, Li L, Wang L, Meng W, Hao Y, Zhu G (2021). Exosome-mediated cellular crosstalk within the tumor microenvironment upon irradiation. Cancer Biol Med.

[CR48] Hei TK, Zhou H, Ivanov VN, Hong M, Lieberman HB, Brenner DJ, Amundson SA, Geard CR (2008). Mechanism of radiation-induced bystander effects: a unifying model. J Pharm Pharmacol.

[CR49] Heller EA, Liu E, Tager AM (2006). Chemokine CXCL10 promotes atherogenesis by modulating the local balance of effector and regulatory T cells. Circulation.

[CR50] Hendry JH, Akahoshi M, Wang LS, Lipshultz SE, Stewart FA, Trott KR (2008). Radiation-induced cardiovascular injury. Radiat Environ Biophys.

[CR51] Hinzman CP, Baulch JE, Mehta KY (2019). Plasma-derived extracellular vesicles yield predictive markers of cranial irradiation exposure in mice. Sci Rep.

[CR52] Hoorelbeke D, Decrock E, De Smet M, De Bock M, Descamps B, Van Haver V (2020). Cx43 channels and signaling via IP3/Ca(2+), ATP, and ROS/NO propagate radiation-induced DNA damage to non-irradiated brain microvascular endothelial cells. Cell Death Dis.

[CR53] Howell R (2012). Second primary cancers and cardiovascular disease after radiation therapy. NCRP report no 170. Med Phys.

[CR54] Howell R, Scarboro S, Kry S, Yaldo D (2010). Accuracy of out-of-field dose calculations by a commercial treatment planning system. Phys Med Biol.

[CR55] Huang S, Che J, Chu Q, Zhang P (2020). The role of NLRP3 inflammasome in radiation-induced cardiovascular injury. Front Cell Dev Biol.

[CR56] Jella KK, Rani S, O'Driscoll L, McClean B, Byrne HJ, Lyng FM (2014). Exosomes are involved in mediating radiation induced bystander signaling in human keratinocyte cells. Radiat Res.

[CR57] Joosten A, Matzinger O, Jeanneret-Sozzi W, Bochud F, Moeckli R (2013). Evaluation of organ-specific peripheral doses after 2-dimensional, 3-dimensional and hybrid intensity modulated radiation therapy for breast cancer based on Monte Carlo and convolution/superposition algorithms: implications for secondary cancer risk assessment. Radiother Oncol.

[CR58] Kadhim M, Tuncay Cagatay S, Elbakrawy EM (2022). Non-targeted effects of radiation: a personal perspective on the role of exosomes in an evolving paradigm. Int J Radiat Biol.

[CR59] Käsmann L, Dietrich A, Staab-Weijnitz CA, Manapov F, Behr J, Rimner A, Jeremic B, Senan S, De Ruysscher D, Lauber K, Belka C (2020). Radiation-induced lung toxicity—cellular and molecular mechanisms of pathogenesis, management, and literature review. Radiat Oncol.

[CR60] Kis D, Persa E, Szatmári T, Antal L, Bóta A, Csordás IB, Hargitai R, Jezsó B, Kis E, Mihály J, Sáfrány G, Varga Z, Lumniczky K (2020). The effect of ionising radiation on the phenotype of bone marrow-derived extracellular vesicles. Br J Radiol.

[CR61] Kis D, Csordás IB, Persa E, Jezsó B, Hargitai R, Szatmári T, Sándor N, Kis E, Balázs K, Sáfrány G, Lumniczky K (2022). Extracellular vesicles derived from bone marrow in an early stage of ionizing radiation damage are able to induce bystander responses in the bone marrow. Cells.

[CR62] Knezevic Z, Stolarczyk L, Bessieres I, Bordy JM, Miljanić S, Olko P (2013). Photon dosimetry methods outside the target volume in radiation therapy: Optically stimulated luminescence (OSL), thermoluminescence (TL) and radiophotoluminescence (RPL) dosimetry. Rad Meas.

[CR63] Knezevic Z, Ambrozova I, Domingo C, De Saint-Hubert M, Majer M, Martinez-Rovira I, Miljanic S, Mojzeszek N, Porwol P, Ploc O, Romero-Exposito M, Stolarczyk L, Trinkl S, Harrison RM, Olko P (2018). Comparison of response of passive dosimetry systems in scanning proton radiotherapy-a study using paediatric anthropomorphic phantoms. Radiat Prot Dosim.

[CR64] Knezevic Z, Stolarczyk L, Ambrožová I, Caballero-Pacheco Á, Davídková M, De Saint-Hubert M, Carles Domingo M, Jeleń K, Kopeć R, Krzempek D, Majer M, Miljanić M, Mojżeszek N, Teresa Romero-Expósito M, Martínez-Rovira I, Harrison R, Olko P (2022). Out-of-field doses produced by a proton scanning beam inside pediatric anthropomorphic phantoms and their comparison with different photon modalities. Front Oncol.

[CR65] Kong FM, Klein EE, Bradley JD, Mansur DB, Taylor ME, Perez CA, Myerson RJ, Harms WB (2002). The impact of central lung distance, maximal heart distance, and radiation technique on the volumetric dose of the lung and heart for intact breast radiation. Int J Radiat Oncol Biol Phys.

[CR66] Kovalchuk A, Kolb B (2017). Low dose radiation effects on the brain—from mechanisms and behavioral outcomes to mitigation strategies. Cell Cycle.

[CR67] Kovalchuk A, Mychasiuk R, Muhammad A, Hossain S, Ilnytskyy S, Ghose A, Kirkby C, Ghasroddashti E, Kovalchuk O, Kolb B (2016). Liver irradiation causes distal bystander effects in the rat brain and affects animal behaviour. Oncotarget.

[CR68] Kry SF, Bednarz B, Howell RM, Dauer L, Followill D, Klein E, Paganetti H, Wang B, Wuu CS, George XuX (2017). AAPM TG 158: measurement and calculation of doses outside the treated volume from external-beam radiation therapy. Med Phys.

[CR69] Kundrát P, Remmele J, Rennau H, Sebb S, Simonetto C, Eidemüller M, Wolf U, Hildebrandt G (2019). Minimum breast distance largely explains individual variability in doses to contralateral breast from breast-cancer radiotherapy. Radiother Oncol.

[CR70] Kundrát P, Rennau H, Remmele J, Sebb S, Simonetto C, Kaiser JC, Hildebrandt G, Wolf U, Eidemüller M (2022). Anatomy-dependent lung doses from 3D-conformal breast-cancer radiotherapy. Sci Rep.

[CR71] Kwak BR, Mulhaupt F, Veillard N, Gros DB, Mach F (2002). Altered pattern of vascular connexin expression in atherosclerotic plaques. Arterioscler Thromb Vasc Biol.

[CR72] Laugaard Lorenzen E, Christian Rehammar J, Jensen MB, Ewertz M, Brink C (2020). Radiation-induced risk of ischemic heart disease following breast cancer radiotherapy in Denmark, 1977–2005. Radiother Oncol.

[CR73] Li G, Lin H, Tian R, Zhao P, Huang Y, Pang X, Zhao L, Cao B (2018). VEGFR-2 inhibitor apatinib hinders endothelial cells progression triggered by irradiated gastric cancer cells-derived exosomes. J Cancer.

[CR300] Li Z, Yang H, Ye L, Quan R, Chen M (2021) Role of exosomal miRNAs in brain metastasis affected by radiotherapy. Transl Neurosci 12(1):127–137. 10.1515/tnsci-2020-0163 10.1515/tnsci-2020-0163 PMC8012736 33821195

[CR74] Li WB, Saldarriaga Vargas C, Bouvier-Capely C, Andersson M, Madas BG (2022). Heterogeneity of dose distribution in normal tissues for radiopharmaceutical therapy with alpha and beta emitters. Radiat Environ Biophys.

[CR75] Lin L, Kane N, Kobayashi N, Kono EA, Yamashiro JM, Nickols NG, Reiter RE (2021). High-dose per fraction radiotherapy induces both antitumor immunity and immunosuppressive responses in prostate tumors. Clin Cancer Res.

[CR76] Little JB (2006). Cellular radiation effects and the bystander response. Mutat Res.

[CR77] Little MP (2016). Radiation and circulatory disease. Mutat Res.

[CR78] Little MP, Tawn EJ, Tzoulaki I, Wakeford R, Hildebrandt G, Paris F, Tapio S, Elliott P (2008). A systematic review of epidemiological associations between low and moderate doses of ionizing radiation and late cardiovascular effects, and their possible mechanisms. Radiat Res.

[CR79] Lowe D, Roy L, Tabocchini MA, Ruhm W, Wakeford R, Woloschak GE, Laurier D (2022). Radiation dose rate effects: what is new and what is needed. Radiat Environ Biophys.

[CR80] Lumniczky K, Sáfrány G (2015). The impact of radiation therapy on the antitumor immunity: local effects and systemic consequences. Cancer Lett.

[CR81] Lumniczky K, Impens N, Armengol G, Candéias S, Georgakilas A, Hornhardt S, Martin O, Rödel F, Schaue D (2021). Low dose ionizing radiation effects on the immune system. Environ Int.

[CR82] Maier A, Wiedemann J, Rapp F, Papenfuß F, Rödel F, Hehlgans S, Gaipl US, Kraft G, Fournier C, Frey B (2020). Radon exposure-therapeutic effect and cancer risk. Int J Mol Sci.

[CR83] Majer M, Stolarczyk L, De Saint-Hubert M, Kabat D, Knezevic Z, Miljanic S, Mojzeszek N, Harrison R (2017). Out-of-field dose measurements for 3d conformal and intensity modulated radiotherapy of a paediatric brain tumour. Radiat Prot Dosim.

[CR84] Majer M, Ambrožová I, Davıdkova M, De Saint-Hubert M, Kasabasič M, Knezevic Z (2022). Out-of-field doses in pediatric craniospinal irradiations with 3d-CRT, VMAT, and scanning proton radiotherapy: a phantom study. Med Phys.

[CR85] Malla B, Aebersold DM, Dal Pra A (2018). Protocol for serum exosomal miRNAs analysis in prostate cancer patients treated with radiotherapy. J Transl Med.

[CR86] Mancuso M, Pazzaglia S, Tanori M, Hahn H, Merola P, Rebessi S, Atkinson MJ, Di Majo V, Covelli V, Saran A (2004). Basal cell carcinoma and its development: insights from radiation-induced tumors in Ptch1-deficient mice. Cancer Res.

[CR87] Mancuso M, Pasquali E, Leonardi S, Tanori M, Rebessi S, Di Majo V, Pazzaglia S, Toni MP, Pimpinella M, Covelli V, Saran A (2008). Oncogenic bystander radiation effects in Patched heterozygous mouse cerebellum. Proc Natl Acad Sci U S A.

[CR88] Mancuso M, Pasquali E, Leonardi S, Rebessi S, Tanori M, Giardullo P, Borra F, Pazzaglia S, Naus CC, Di Majo V, Saran, (2011). A role of connexin43 and ATP in long-range bystander radiation damage and oncogenesis in vivo. Oncogene.

[CR89] Mancuso M, Pasquali E, Giardullo P, Leonardi S, Tanori M, Di Majo V, Pazzaglia S, Saran A (2012). The radiation bystander effect and its potential implications for human health. Curr Mol Med.

[CR90] Mancuso M, Pasquali E, Braga-Tanaka I, Tanaka S, Pannicelli A, Giardullo P, Pazzaglia S, Tapio S, Atkinson MJ, Saran A (2015). Acceleration of atherogenesis in ApoE−/− mice exposed to acute or low-dose-rate ionizing radiation. Oncotarget.

[CR91] Marquette C, Linard C, Galonnier M, Van Uye A, Mathieu J, Gourmelon P, Clarençon D (2003). IL-1beta, TNFalpha and IL-6 induction in the rat brain after partial-body irradiation: role of vagal afferents. Int J Radiat Biol.

[CR92] Mazonakis M, Damiliakis J (2021). Out-of-field organ doses and associated risk of cancer development following radiation therapy with photons. Phys Med.

[CR93] Miljanić S, Bessieres I, Bordy JM, d'Errico F, Di Fulvio A, Kabat D, Knezevic Z, Olko P, Stolarczyk L, Tana L, Harrison R (2013). Clinical simulations of prostate radiotherapy using BOMAB-like phantoms: results for photons. Rad Meas.

[CR94] Moertl S, Buschmann D, Azimzadeh O, Schneider M, Kell R, Winkler K, Tapio S, Hornhardt S, Merl-Pham J, Pfaffl MW, Atkinson MJ (2020). Radiation exposure of peripheral mononuclear blood cells alters the composition and function of secreted extracellular vesicles. Int J Mol Sci.

[CR95] Mojżeszek N, Farah J, Kłodowska M, Ploc O, Stolarczyk L, Waligórski M (2017). Measurement of stray neutron doses inside the treatment room from a proton pencil beam scanning system. Phys Med.

[CR96] Morgan WF, Sowa MB (2015). Non-targeted effects induced by ionizing radiation: mechanisms and potential impact on radiation induced health effects. Cancer Lett.

[CR97] Mrowczynski OD, Madhankumar AB, Sundstrom JM, Zhao Y, Kawasawa YI, Slagle-Webb B, Mau C, Payne RA, Rizk EB, Zacharia BE, Connor JR (2018). Exosomes impact survival to radiation exposure in cell line models of nervous system cancer. Oncotarget.

[CR98] Mutschelknaus L, Peters C, Winkler K, Yentrapalli R, Heider T, Atkinson MJ (2016). Exosomes derived from squamous head and neck cancer promote cell survival after ionizing radiation. PLoS ONE.

[CR99] Mutschelknaus L, Azimzadeh O, Heider T (2017). Radiation alters the cargo of exosomes released from squamous head and neck cancer cells to promote migration of recipient cells. Sci Rep.

[CR100] Nakaoka A, Nakahana M, Inubushi S, Akasaka H, Salah M, Fujita Y, Kubota H, Hassan M, Nishikawa R, Mukumoto N, Ishihara T, Miyawaki D, Sasayama T, Sasaki R (2021). Exosome-mediated radiosensitizing effect on neighboring cancer cells via increase in intracellular levels of reactive oxygen species. Oncol Rep.

[CR101] NCRP (2011) Second primary cancers and cardiovascular disease after radiation therapy. NCRP Report No 170 National Council on Radiation Protection and Measurements, Bethesda, Maryland

[CR102] Newhauser WD, Schneider C, Wilson L, Shrestha S, Donahue W (2018). A review of analytical models of stray radiation exposures from photon- and proton-beam radiotherapies. Radiat Prot Dosim.

[CR103] Niel G, D'Angelo G, Raposo G (2018). Shedding light on the cell biology of extracellular vesicles. Nat Rev Mol Cell Biol.

[CR104] Nikitaki Z, Mavragani IV, Laskaratou DA, Gika V, Moskvin VP, Theofilatos K (2016). Systemic mechanisms and effects of ionizing radiation: a new ‘old’ paradigm of how the bystanders and distant can become the players. Semin Cancer Biol.

[CR105] Ohshima Y, Tsukimoto M, Harada H, Kojima S (2012). Involvement of connexin43 hemichannel in ATP release after γ-irradiation. J Radiat Res.

[CR106] Park H, Paganetti H, Schuemann J, Jia X, Min CH (2021). Monte Carlo methods for device simulations in radiation therapy. Phys Med Biol.

[CR107] PASSOS (2022) Personalised assessment of late health risks after exposure to ionising radiation and guidance for radiation applications in medicine. https://passos.helmholtz-muenchen.de Accessed 27 Jan 2022

[CR108] Pazzaglia S, Mancuso M, Atkinson MJ, Tanori M, Rebessi S, Majo VD, Covelli V, Hahn H, Saran A (2002). High incidence of medulloblastoma following X-ray-irradiation of newborn Ptc1 heterozygous mice. Oncogene.

[CR109] Pazzaglia S, Tanno B, Antonelli F, Giardullo P, Babini G, Subedi P, Azimzadeh O, Khan ZN, Oleksenko K, Metzger F, Toerne CV, Traynor D, Medipally D, Meade AD, Kadhim M, Lyng FM, Tapio S, Saran A, Mancuso M (2021). Out-of-field hippocampus from partial-body irradiated mice displays changes in multi-omics profile and defects in neurogenesis. Int J Mol Sci.

[CR110] Pazzaglia S, Tanno B, De Stefano I, Giardullo P, Leonardi S, Merla C, Babini G, Tuncay Cagatay S, Mayah A, Kadhim M, Lyng F, C von Toerne, Khan ZK, Subedi P, Tapio S, Saran A, Mancuso M (2022) Micro-RNA and proteomic profiles of plasma derived exosomes from irradiated mice reveal molecular changes preventing apoptosis in neonatal cerebellum. Int J Mol Sci **(in press)** 10.3390/ijms23042169 PMC8878539 35216284

[CR111] Persa E, Szatmári T, Sáfrány G, Lumniczky K (2018). In vivo irradiation of mice induces activation of dendritic cells. Int J Mol Sci.

[CR112] Pouget JP, Georgakilas AG, Ravanat JL (2018). Targeted and off-target (bystander and abscopal) effects of radiation therapy: redox mechanisms and risk/benefit analysis. Antiox Redox Signal.

[CR113] Preston DL, Ron E, Tokuoka S, Funamoto S, Nishi N, Soda M, Mabuchi K, Kodama K (2007). Solid cancer incidence in atomic bomb survivors: 1958–1998. Radiat Res.

[CR114] Rabin BM, Shukitt-Hale B, Carrihill-Knoll KL, Gomes SM (2014). Comparison of the effects of partial- or whole-body exposures to ^16^O particles on cognitive performance in rats. Radiat Res.

[CR115] Rabin BM, Poulose SM, Bielinski DF, Shukitt-Hale B (2019). Effects of head-only or whole-body exposure to very low doses of 4He (1000 MeV/n) particles on neuronal function and cognitive performance. Life Sci Space Res (amst).

[CR116] Ramadan R, Vromans E, Anang DC (2019). Single and fractionated ionizing radiation induce alterations in endothelial connexin expression and channel function. Sci Rep.

[CR117] Ramadan R, Vromans E, Anang DC, Goetschalckx I, Hoorelbeke D, Decrock E (2020). Connexin43 hemichannel targeting with TAT-Gap19 alleviates radiation-induced endothelial cell damage. Front Pharmacol.

[CR118] Ramadan R, Baatout S, Aerts A, Leybaert L (2021). The role of connexin proteins and their channels in radiation-induced atherosclerosis. Cell Mol Life Sci.

[CR119] Ramadan R, Claessens M, Cocquyt E, Mysara M, Decrock E, Baatout S (2021). X-irradiation induces acute and early term inflammatory responses in atherosclerosis-prone ApoE−/− mice and in endothelial cells. Mol Med Rep.

[CR120] RKI (2022) German Centre for Cancer Registry Data. Database Query with estimates for cancer incidence, prevalence and survival in Germany, based on data of the population based cancer registries. Robert Koch Institute. https://www.krebsdaten.de/Krebs/EN/Content/Cancer_sites/Breast_cancer/breast_cancer_node.html Accessed 27 Jan 2022

[CR121] Rödel F, Frey B, Manda K, Hildebrandt G, Hehlgans S, Keilholz L, Seegenschmiedt HM, Gaipl US, Rödel C (2012). Immunomodulatory properties and molecular effects in inflammatory diseases of low-dose x-irradiation. Front Oncol.

[CR122] Ruben JD, Lancaster CM, Jones P, Smith RL (2011). A comparison of out-of-field dose and its constituent components for intensity-modulated radiation therapy versus conformal radiation therapy: implications for carcinogenesis. Int J Rad Oncol Biol Phys.

[CR123] Ruhm W, Harrison RM (2020). High CT doses return to the agenda. Radiat Environ Biophys.

[CR124] Sachs RK, Brenner DJ (2005). Solid tumor risks after high doses of ionizing radiation. Proc Natl Acad Sci U S A.

[CR125] Sayah R, Farah J, Donadille L, Hérault J, Delacroix S, De Marzi L (2014). Secondary neutron doses received by paediatric patients during intracranial proton therapy treatments. J Radiol Prot.

[CR126] Schneider U (2009). Mechanistic model of radiation-induced cancer after fractionated radiotherapy using the linear-quadratic formula. Med Phys.

[CR127] Schneider U, Walsh L (2017). Risk of secondary cancers: Bridging epidemiology and modeling. Phys Med.

[CR128] Schneider U, Zwahlen D, Ross D, Kaser-Hotz B (2005). Estimation of radiation-induced cancer from three-dimensional dose distributions: concept of organ equivalent dose. Int J Rad Oncol Biol Phys.

[CR129] Schneider U, Walsh L, Newhauser W (2018). Tumour size can have an impact on the outcomes of epidemiological studies on second cancers after radiotherapy. Radiat Environ Biophys.

[CR130] Schultz-Hector S, Trott KR (2007). Radiation-induced cardiovascular diseases: is the epidemiologic evidence compatible with the radiobiologic data. Int J Radiat Oncol Biol Phys.

[CR131] Shi X, Shiao SL (2018). The role of macrophage phenotype in regulating the response to radiation therapy. Transl Res.

[CR132] Shuryak I, Hahnfeldt P, Hlatky L, Sachs RK, Brenner DJ (2009). A new view of radiation-induced cancer: integrating short- and long-term processes. Part I: approach. Radiat Environ Biophys.

[CR133] Shuryak I, Hahnfeldt P, Hlatky L, Sachs RK, Brenner DJ (2009). A new view of radiation-induced cancer: integrating short- and long-term processes. Part II: second cancer risk estimation. Radiat Environ Biophys.

[CR134] Simonetto C, Rennau H, Remmele J, Sebb S, Kundrát P, Eidemüller M, Wolf U, Hildebrandt G (2019). Exposure of remote organs and associated cancer risks from tangential and multi-field breast cancer radiotherapy. Strahlenther Onkol.

[CR135] Simonetto C, Eidemüller M, Gaasch A, Pazos M, Schönecker S, Reitz D, Kääb S, Braun M, Harbeck N, Niyazi M, Belka C, Corradini S (2019). Does deep inspiration breath-hold prolong life? Individual risk estimates of ischaemic heart disease after breast cancer radiotherapy. Radiother Oncol.

[CR136] Simonetto C, Wollschläger D, Kundrát P, Ulanowski A, Becker J, Castelletti N, Güthlin D, Shemiakina E, Eidemüller M (2021). Estimating long-term health risks after breast cancer radiotherapy: merging evidence from low and high doses. Radiat Environ Biophys.

[CR137] Sprung CN, Forrester HB, Siva S, Martin OA (2015). Immunological markers that predict radiation toxicity. Cancer Lett.

[CR138] Stewart FA, Akleyev AV, Hauer-Jensen M, Hendry JH, Kleiman NJ (2012). ICRP statement on tissue reactions and early and late effects of radiation in normal tissues and organs–threshold doses for tissue reactions in a radiation protection context. Ann ICRP.

[CR139] Stolarczyk L, Trinkl S, Romero-Exposito M, Mojzeszek N, Ambrozova I, Domingo C, Davidkova M, Farah J, Klodowska M, Knezevic Z, Liszka M, Majer M, Miljanic S, Ploc O, Schwarz M, Harrison RM, Olko P (2018). Dose distribution of secondary radiation in a water phantom for a proton pencil beam-EURADOS WG9 intercomparison exercise. Phys Med Biol.

[CR140] Storozynsky Q, Hitt MM (2020). The impact of radiation-induced DNA damage on cGAS-STING-mediated immune responses to cancer. Int J Mol Sci.

[CR141] Szatmári T, Kis D, Bogdándi EN, Benedek A, Bright S, Bowler D, Persa E, Kis E, Balogh A, Naszályi LN, Kadhim M, Sáfrány G, Lumniczky K (2017). Extracellular vesicles mediate radiation-induced systemic bystander signals in the bone marrow and spleen. Front Immunol.

[CR142] Szatmári T, Hargitai R, Sáfrány G, Lumniczky K (2019). Extracellular vesicles in modifying the effects of ionizing radiation. Int J Mol Sci.

[CR143] Taffoni C, Steer A, Marines J, Chamma H, Vila IK, Laguette N (2021). Nucleic acid immunity and DNA damage response: new friends and old foes. Front Immunol.

[CR144] Tavakolian FV, Mohammadi M, Hassanshahi G, Khorramdelazad H, Khanamani FS, Mirzaei M (2017). Serum CXCL10 and CXCL12 chemokine levels are associated with the severity of coronary artery disease and coronary artery occlusion. Int J Cardiol.

[CR145] Théry C (2018). Minimal information for studies of extracellular vesicles 2018 (MISEV2018): a position statement of the International Society for Extracellular Vesicles and update of the MISEV2014 guidelines. J Extracell Vesicles.

[CR146] Tortolici F, Vumbaca S, Incocciati B, Dayal R, Aquilano K, Giovanetti A, Rufini S (2021). Ionizing radiation-induced extracellular vesicle release promotes AKT-associated survival response in SH-SY5Y neuroblastoma cells. Cells.

[CR147] Tsukimoto M, Homma T, Ohshima Y, Kojima S (2010). Involvement of purinergic signaling in cellular response to gamma radiation. Radiat Res.

[CR148] Tuncay CS, Mayah A, Mancuso M, Giardullo P, Pazzaglia S, Saran A, Daniel A, Traynor D, Meade AD, Lyng F, Tapio S, Kadhim M (2020). Phenotypic and functional characteristics of exosomes derived from irradiated mouse organs and their role in the mechanisms driving non-targeted effects. Int J Mol Sci.

[CR149] Vu BJ, Allodji RS, Mege JP, BeldjoudiG SF, Chavaudra J, Deutsch E, Vathaire F, Bernier V, Carrie C, Lefkopoulos D, Diallo I (2017). A review of uncertainties in radiotherapy dose reconstruction and their impacts on dose-response relationships. J Radiol Prot.

[CR150] Wagner T, Traxler D, Simader E, Beer L, Narzt MS, Gruber F, Madlener S, Laggner M, Erb M, Vorstandlechner V, Gugerell A, Radtke C, Gnecchi M, Peterbauer A, Gschwandtner M, Tschachler E, Keibl C, Slezak P, Ankersmit HJ, Mildner M (2018). Different pro-angiogenic potential of γ-irradiated PBMC-derived secretome and its subfractions. Sci Rep.

[CR151] Walle T, Martinez Monge R, Cerwenka A, Ajona D, Melero I, Lecanda F (2018). Radiation effects on antitumor immune responses: current perspectives and challenges. Ther Adv Med Oncol.

[CR152] Wang R, Zhou T, Liu W, Zuo L (2018). Molecular mechanism of bystander effects and related abscopal/cohort effects in cancer therapy. Oncotarget.

[CR153] Wijerathne H, Langston JC, Yang Q, Sun S, Miyamoto C, Kilpatrick LE (2021). Mechanisms of radiation-induced endothelium damage: emerging models and technologies. Radiother Oncol.

[CR154] Wochnik A, Stolarczyk L, Ambrožová I, Davıdkova ´ ´ M, De Saint-Hubert M, Domanski S, (2021). Out-of-field doses for scanning proton radiotherapy of shallowly located paediatric tumours—a comparison of range shifter and 3D printed compensator. Phys Med Biol.

[CR155] Xu X, Bednarz B, Paganetti H (2008). A review of dosimetry studies on external beam radiation treatment with respect to second cancer induction. Phys Med Biol.

[CR156] Xu X, Li Z, Gao W (2011). Growth differentiation factor 15 in cardiovascular diseases: from bench to bedside. Biomarkers.

[CR157] Xu S, Wang J, Ding N, Hu W, Zhang X, Wang B (2015). Exosome-mediated microRNA transfer plays a role in radiation-induced bystander effect. RNA Biol.

[CR158] Yu X, Harris SL, Levine AJ (2006). The regulation of exosome secretion: a novel function of the p53 protein. Cancer Res.

[CR159] Zhang R, Mirkovic D, Newhauser WD (2015). Visualization of risk of radiogenic second cancer in the organs and tissues of the human body. Radiat Oncol.

[CR160] Zhang MdJ, Zhang MdL, Yang MdY, Liu MdQ, Ma MdH, Huang MdA, Zhao MdY, Xia MdZ, Liu MdT, Wu MdG (2021). Polymorphonuclear-MDSCs facilitate tumor regrowth after radiation by suppressing CD8+ T cells. Int J Radiat Oncol Biol Phys.

[CR161] Zheng Y, Liu L, Chen C, Ming P, Huang Q, Li C, Cao D, Xu X, Ge W (2017). The extracellular vesicles secreted by lung cancer cells in radiation therapy promote endothelial cell angiogenesis by transferring miR-23a. PeerJ.

[CR162] Zhou M, Yang M, Zhang J (2021). Immunogenic cell death induction by ionizing radiation. Front Immunol.

[CR163] Zhu M, Yang M, Zhang J, Yin Y, Fan X, Zhang Y, Qin S, Zhang H, Yu F (2021). Immunogenic cell death induction by ionizing radiation. Front Immunol.

