# Correction to: Out‑of‑field effects: lessons learned from partial body exposure

**DOI:** 10.1007/s00411-022-01003-2

**Published:** 2022-10-17

**Authors:** S. Pazzaglia, M. Eidemüller, K. Lumniczky, M. Mancuso, R. Ramadan, L. Stolarczyk, S. Moertl

**Affiliations:** 1 Laboratory of Biomedical Technologies, ENEA CR-Casaccia, Via Anguillarese 301, 00123 Rome, Italy; 2 Institute of Radiation Medicine, Helmholtz Zentrum München, Ingolstädter Landstraße 1, 85764 Neuherberg, Germany; 3 Department of Radiobiology and Radiohygiene, Unit of Radiation Medicine, National Public Health Centre, Albert Florian u. 2-6, 1097 Budapest, Hungary; 4 Radiobiology Unit, Belgian Nuclear Research Centre (SCK CEN), Mol, Belgium; 5 Danish Centre for Particle Therapy, Palle Juul-Jensens Boulevard 25, 8200 Aarhus N, Denmark; 6 Federal Office for Radiation Protection, Ingolstädter Landstr. 1, 85764 Oberschleißheim, Germany

## Correction to: Radiation and Environmental Biophysics 10.1007/s00411-022-00988-0

Editor would like to update DOI of the references.

The original article has been corrected.
