# Correction to: Out‑of‑field effects: lessons learned from partial body exposure

**DOI:** 10.1007/s00411-022-01006-z

**Published:** 2022-12-06

**Authors:** S. Pazzaglia, M. Eidemüller, K. Lumniczky, M. Mancuso, R. Ramadan, L. Stolarczyk, S. Moertl

**Affiliations:** 1 Laboratory of Biomedical Technologies, ENEA CR-Casaccia, Via Anguillarese 301, 00123 Rome, Italy; 2 Institute of Radiation Medicine, Helmholtz Zentrum München, Ingolstädter Landstraße 1, 85764 Neuherberg, Germany; 3 Department of Radiobiology and Radiohygiene, Unit of Radiation Medicine, National Public Health Centre, Albert Florian u. 2-6, 1097 Budapest, Hungary; 4 Radiobiology Unit, Belgian Nuclear Research Centre (SCK CEN), Mol, Belgium; 5 Danish Centre for Particle Therapy, Palle Juul-Jensens Boulevard 25, 8200 Aarhus N, Denmark; 6 Federal Office for Radiation Protection, Ingolstädter Landstr. 1, 85764 Oberschleißheim, Germany

**Correction to: Radiation and Environmental Biophysics (2022) 61:485–504** 10.1007/s00411-022-00988-0

The article “Out‑of‑field effects: lessons learned from partial body exposure”, written by S. Pazzaglia, M. Eidemüller, K. Lumniczky, M. Mancuso, R. Ramadan, L. Stolarczyk and S. Moertl, was originally published Online First without Open Access. After publication in volume 61, issue 4, page 485–504 the author decided to opt for Open Choice and to make the article an Open Access publication. Therefore, the copyright of the article has been changed to © The Author(s) 2022 and the article is forthwith distributed under the terms of the Creative Commons Attribution 4.0 International License, which permits use, sharing, adaptation, distribution and reproduction in any medium or format, as long as you give appropriate credit to the original author(s) and the source, provide a link to the Creative Commons licence, and indicate if changes were made. The images or other third party material in this article are included in the article’s Creative Commons licence, unless indicated otherwise in a credit line to the material. If material is not included in the article’s Creative Commons licence and your intended use is not permitted by statutory regulation or exceeds the permitted use, you will need to obtain permission directly from the copyright holder. To view a copy of this licence, visit http://creativecommons.org/licenses/by/4.0.

The original article has been corrected.

